# Climbing up or falling down: Narcissism predicts physiological sensitivity to social status in children and their parents

**DOI:** 10.1111/desc.13062

**Published:** 2020-11-25

**Authors:** Stathis Grapsas, Jaap J. A. Denissen, Hae Yeon Lee, Peter A. Bos, Eddie Brummelman

**Affiliations:** ^1^ Department of Developmental Psychology Tilburg University Tilburg the Netherlands; ^2^ Department of Developmental Psychology Utrecht University Utrecht the Netherlands; ^3^ Department of Psychology Stanford University Stanford CA USA; ^4^ Institute of Education and Child Studies Leiden University Leiden the Netherlands; ^5^ Research Institute of Child Development and Education University of Amsterdam Amsterdam the Netherlands

**Keywords:** childhood narcissism, facial electromyography, physiological sensitivity, social status

## Abstract

Children's narcissism may be rooted in sensitivity to *social status* (i.e., prominence, respect, and influence in a social group), and this sensitivity might be shared with parents. Testing this idea, a randomized experiment examined how children with high narcissism levels and their parents respond to gains and losses of social status. On a simulated social media platform, children (*N* = 123, ages 8–13) competed with fictitious peers for status and were randomly assigned to gain or lose status. Unbeknownst to children, parents viewed the course of the task. Children's and parents' affective reactions during the task were measured with facial electromyography, which detects spontaneous facial muscle activity linked to positive affect (i.e., zygomaticus major activity, involved in smiling) and negative affect (i.e., corrugator supercilii activity, involved in frowning). Children with higher narcissism levels showed steeper increases in negative affect during status loss and steeper increases in both positive and negative affect during status gain. Their parents mirrored the steeper increase in positive affect during their child's status gain, but they did not mirror the increase in negative affect. These results suggest that children with high narcissism levels and their parents show intensified affective‐motivational responses to children's status‐relevant experiences. These responses may be transmitted from one generation to the other (e.g., genetically or through parent–child socialization).

## Graphical abstract

Children’s narcissism may be rooted in sensitivity to social status (i.e., prominence, respect, and social influence), and this sensitivity might be shared with parents. In a social media environment, we tracked children’s and parents’ spontaneous affective responses to gains and losses of social status using facial electromyography. We found that children with high narcissism levels as well as their parents showed intensified affective responses to children’s status gains and losses.

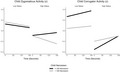

**Research Highlights**

- A randomized experiment examined how children with high narcissism levels and their parents respond to gain and loss of social status
- Status gain and loss was induced using a simulated social media platform, where children competed with peers for popularity
- Affective reactions were indexed using facial electromyography: Children with high narcissism levels as well as their parents showed intensified affective responses to status gain and loss
- Results suggest that childhood narcissism may be underpinned by status sensitivity and that this sensitivity is transmitted across generations

## 1 INTRODUCTION

From a young age, children are concerned about *social status*. Status indicates children's position within a social hierarchy (Anderson et al., 2015) and is often reflected in their popularity (i.e., social visibility, importance, and influence; Lease et al., 2002). Although the status motive is universal (Anderson et al., 2015), there might be individual differences in children's sensitivity to status gains and losses (McClelland, 1987). We propose that children's status sensitivity is positively associated with narcissism. We hypothesized that children with higher narcissism levels would show intensified positive and negative affective responsiveness to status gains and losses, and that so would their parents. We tested this idea in a randomized experiment, using facial electromyography (fEMG) to track children's and parents' affective responses (e.g., smiling, frowning) to children's status gains and losses.

### 1.1 Narcissism and status sensitivity

Narcissism is a personality trait that emerges in childhood and is characterized by feelings of superiority, sense of entitlement, and desire for respect and admiration (Thomaes, Stegge, et al., 2008). Narcissism is not a disorder but a normally distributed personality trait, with no cut‐off that separates “non‐narcissists” from “narcissists” (Foster & Campbell, 2007). Certain measures of narcissism are related to mental health problems and problematic social relationships (e.g., internalizing problems, aggression, and bullying; Barry & Malkin, 2010; Reijntjes et al., 2016). Extreme levels of narcissism can develop into narcissistic personality disorder (Miller & Campbell, 2008), which is rarely diagnosed before adulthood (American Psychiatric Association, 2013).

We propose that childhood narcissism is characterized by heightened sensitivity to status. Personality traits may be coherent sets of strategies that serve specific motives, such as status (Denissen & Penke, 2008). Motivation often operates via affect, which signals pleasure and displeasure when individuals fulfill or fail to fulfill their motives (Berridge & Winkielman, 2003). According to motive disposition theory, individuals differ in their tendency to derive pleasure or displeasure from motive‐relevant experiences (McClelland, 1987). Traits, then, might have unique affective‐motivational contingencies (e.g., "*If* people admire me, *then* I feel great"; Mischel & Shoda, 1995). Accordingly, narcissism might be underpinned by a strong status motive, giving rise to a heightened sensitivity to status gains and losses (Morf & Rhodewalt, 2001; Zeigler‐Hill et al., 2018). This status sensitivity might underlie narcissism from childhood (Grapsas et al., 2020).

Although this proposal has never been tested directly, indirect evidence supports it. Narcissism tends to emerge in middle‐to‐late childhood (Thomaes & Brummelman, 2016), when children start pursuing status more vigorously (Hawley, 1999). From this age, children with high narcissism levels tend to desire and seek status (Ojanen et al., 2012; Thomaes, Stegge, et al., 2008). To gain status, they may brag, show off, and try to be the center of attention (Reijntjes et al., 2016; Thomaes & Brummelman, 2016), often emerging as leaders in peer groups (Brummelman et al., in press). When losing status, however, they may lash out against competitors (Thomaes, Bushman, et al., 2008). Such motive‐relevant behaviors are often fueled by motive‐relevant affective responses (Dufner et al., 2015). We theorize that status sensitivity can reveal itself in heightened positive affect in response to status gains and heightened negative affect in response to status losses.

Children with high narcissism levels might acquire their status sensitivity partly through their parents. Given the heritability of narcissism (Vernon et al., 2008), status sensitivity might be genetically transmitted. Given the malleability of narcissism (Brummelman & Sedikides, 2020), parents might socialize status sensitivity. Status sensitivity might be reinforced by parental overvaluation—parents seeing and treating their children as more special and entitled, while pressuring them to stand out (Brummelman, Thomaes, Nelemans, Orobio de Castro, Overbeek, et al., 2015). Both perspectives suggest that parents of children with high narcissism levels exhibit heightened status sensitivity. We theorize that parents' status sensitivity can reveal itself in their affective responses to their children's status gains and losses.

### 1.2 Capturing status sensitivity

Children with high narcissism levels might be unable or unwilling to report their negative affective responses. They often do not report negative affect, even when their physiological responses suggest they do experience it (Brummelman et al., 2018; Cascio et al., 2015). We, therefore, used fEMG to track children's and parents' spontaneous affective responses to status gains and losses. Zygomaticus major muscle activity is involved in smiling, reflecting positive affect, whereas corrugator supercilii muscle activity is involved in frowning, reflecting negative affect (Cacioppo et al., 1986). Because these spontaneous muscle contractions are difficult to suppress, they may reliably capture ongoing affective responses (Barrett et al., 2019).

### 1.3 Present study

In a randomized experiment, we investigated how children with higher narcissism levels and their parents affectively responded to the child's status gain and loss. We focused on late childhood when individual differences in narcissism first arise (Thomaes & Brummelman, 2016). We first assessed children's narcissism and parents' overvaluation levels. Children performed an ecologically valid social media task, wherein they were randomly assigned to gain or lose status among same‐age fictitious peers. Unbeknownst to children, parents watched this task on another screen. We hypothesized that children with higher narcissism levels and their parents would exhibit intensified positive affect (zygomaticus activity) in response to status gain and intensified negative affect (corrugator activity) in response to status loss.

## 2 METHOD

### 2.1 Participants

Participants were 123 children (99% Dutch origin; 55% girls) aged 8–13 (*M* = 10.11, *SD* = 1.42) and one of their parents (92% Dutch origin; 55% females) aged 34–58 (*M* = 44.22, *SD* = 5.42). The research was part of Science Live, a research program that enables scientists to recruit visitors to science museum NEMO (Amsterdam, the Netherlands) as participants. Parents provided active informed consent for themselves and their children. Children provided assent. Study procedures were approved by the Ethics Review Board of Tilburg University School of Social and Behavioral Sciences. Post hoc power analyses with 1,000 Monte Carlo simulations (simr package Version 1.0.5; Green & MacLeod, 2016) showed that the final sample size had sufficient power (above 80% in all but one cases, *α* = 0.05, two‐tailed) to detect the observed effect sizes of the highest‐order significant interactions (Supplementary Material).

### 2.2 Procedure and measures

We reported all measures analyzed for this article's research questions (for all study measures, see Supplementary Material).

#### 2.2.1 Narcissism

Children completed the 10‐item Childhood Narcissism Scale, which has a unidimensional structure and is normally distributed (Thomaes, Bushman, et al., 2008; Thomaes, Stegge, et al., 2008). Items (e.g., “I am a very special person”) were rated on 4‐point scales (0 = *not at all true*, 3 = *completely true*). Responses were averaged across items (*M* = 1.21, *SD* = 0.45, *α* = 0.73). For validation purposes, we also administered the Narcissistic Admiration and Rivalry Questionnaire Short Scale (Back et al., 2013), an alternative narcissism measure that we adapted for use with children. We present results for this scale in the Supplementary Material.

#### 2.2.2 Parental overvaluation

Parents completed the 7‐item Parental Overvaluation Scale, which has the unidimensional structure and is normally distributed (Brummelman, Thomaes, Nelemans, Orobio de Castro, & Bushman, 2015). Items (e.g., “My child is more special than other children”) were answered on 4‐point scales (0 = *not at all true*, 3 = *completely true*). Responses were averaged across items (*M* = 1.32, *SD* = 0.51, *α* = 0.75).

#### 2.2.3 Social media task

Children participated in an online social media task (adapted from Lee et al., 2020; Wolf et al., 2015). Parents secretly viewed the task on another screen.

To emulate real‐life status competition, the task was framed as an online popularity contest (Supplementary Material). Children created a public profile and interacted online with 11 fictitious peers to collect likes for being “special and exceptional,” concepts that overlap with children's representations of high‐status, popular individuals (Gülgöz & Gelman, 2017; Vaughn & Waters, 1981). Likes were visible below each profile, and contestants' placement in the popularity hierarchy was shown on a ranking board. Children were randomly assigned to a *high‐status* or *low‐status* condition. Children in the high‐status condition (*n* = 41) received nine likes out of 11 over time. Reflecting a high yet believable level of status, children progressively rose in popularity, ending up in the second‐highest position. Children in the low‐status condition (*n* = 42) received only two likes out of 11, progressively dropping to, and remaining at, the bottom of the ranking. The likes that fictitious peers received remained constant across conditions. The interaction lasted for three minutes, with time visibly counted down.

Our study also included a third condition (*n* = 40), intended to be neutral. In this condition, children were informed that they would compete for popularity, but there was no option to give or receive likes. Debriefing revealed that children in this condition felt confused and frustrated during the task, as they could not understand how popularity was being determined. For this reason, we excluded this condition from analyses.

#### 2.2.4 fEMG recordings

We measured children's and parents' affective reactions via fEMG during a baseline, when participants viewed a waiting screen (5 s), and during the task (180 s). We recorded muscle activity of the zygomaticus major (contracted when smiling, reflecting positive affect) and the corrugator supercilii (contracted when frowning, reflecting negative affect). Muscle activity was filtered (van Boxtel, 2010), rectified, and aggregated per second (Supplementary Material).

#### 2.2.5 Manipulation check

After the task, children indicated whether they were popular during the task (1 = *not at all true*, 4 = *completely true*) and how many more likes they received compared to their peers (1 = *much fewer likes*, 5 = *many more likes*). Parents indicated whether their child was popular during the task (1 = *not at all true*, 4 = *completely true*) and how many more likes their child received compared to its peers (1 = *much fewer likes*, 5 = *many more likes*). Participants could also respond “*I do not know*” (coded as missing in analyses).

### 2.3 Analytic strategy

Analyses were conducted in R (Version 3.6.1; R Core Team, 2019). Within participants, we winsorized extreme muscle activity values (i.e., we substituted values exceeding the 95%‐quantile with the value of the 95%‐quantile). Continuous variables were z‐standardized. We used the lme4 package (Version 3.6.3; Bates et al., 2015) to run random‐intercept multilevel models, separately per muscle (zygomaticus, corrugator). Dependent variables were nested within individuals and were regressed on time (continuous within‐subjects), condition (between‐subjects; 0 = low status, 1 = high status), children's narcissism or parental overvaluation (continuous between‐subjects), and their two‐ and three‐way interactions. *p* values were extracted with the lmerTest package (Version 3.1.1; Kuznetsova et al., 2017). Results were identical when *p* values were obtained using model comparison (Supplementary Material). Significant interactions were broken down by condition and probed using simple slopes (Aiken & West, 1991).

## 3 RESULTS

### 3.1 Preliminary analyses

Descriptive statistics and correlations are presented in Table 1. Consistent with prior findings (Brummelman, Thomaes, Nelemans, Orobio de Castro, Overbeek, et al., 2015; Derry, 2018; Nguyen & Shaw, 2020), there was a modest, although statistically non‐significant, positive association of children's narcissism with parental overvaluation.

**TABLE 1:** Means, standard deviations, and correlations for main variables in children and parents

	*M*	*SD*	Correlations				
			2.	3.	4.	5.	6.
1. Children's narcissism	1.25	0.45	0.14	0.05	0.01	−0.01	−0.07
Low status	1.21	0.49	0.16	0.12	−0.02	0.05	−0.04
High status	1.28	0.42	0.10	−0.05	0.08	−0.05	−0.19
2. Parental overvaluation	1.31	0.54		0.00	−0.03	−0.01	−0.07
Low status	1.42	0.55		−0.04	−0.06	0.08	0.00
High status	1.21	0.51		0.18	0.09	−0.07	−0.27
3. Child corrugator activity (*z*)	0.00	1.00			0.11***	0.02	0.04***
Low status	0.19	1.22			0.01	−0.02	0.04**
High status	−0.19	0.67			0.21***	0.07***	0.04***
4. Child zygomaticus activity (*z*)	0.00	1.00				−0.01	0.01
Low status	0.12	1.23				−0.02	0.04***
High status	−0.12	0.69				0.00	−0.03**
5. Parent corrugator activity (*z*)	0.00	1.00					−0.02*
Low status	−0.03	0.75					−0.01
High status	0.03	1.21					−0.04***
6. Parent zygomaticus activity (*z*)	0.00	1.00					
Low status	0.08	1.35					
High status	−0.09	0.42					

Correlations between muscles are within‐person.

* *p* < 0.05,

** *p* < 0.01,

*** *p* < 0.001.

There were no condition differences in children's age, narcissism, or sex, nor in parents' age, overvaluation, or sex, *ps* ≥ 0.080, indicating successful random assignment. Compared to children in the low‐status condition, children in the high‐status condition reported being more popular, *t*(64) = 9.44, *p* < 0.001 (low status: *M* = 1.76, *SD* = 0.79; high status: *M* = 3.33, *SD* = 0.54) and receiving more likes, *t*(68) = 7.27, *p* < 0.001 (low status: *M* = 2.27, *SD* = 1.26; high status: *M* = 4.12, *SD* = 0.78). Compared to parents in the low‐status condition, parents in the high‐status condition reported their child being more popular, *t*(79) = 11.60, *p* < 0.001 (low status: *M* = 1.50, *SD* = 0.71; high status: *M* = 3.23, *SD* = 0.63) and receiving more likes, *t*(74) = 12.10, *p* < 0.001 (low status: *M* = 1.31, *SD* = 0.83; high status: *M* = 3.65, *SD* = 0.86). Thus, the manipulation appeared effective.

### 3.2 Children's affective responses

Our first set of analyses examined children's sensitivity to status gains and losses.

#### 3.2.1 Zygomaticus activity

We tested the hypothesis that children's narcissism would be associated with increased zygomaticus activity when children gained status (Table 2). There were no effects of condition or narcissism, but there was an effect of time, with zygomaticus activity decreasing over the course of the experiment. There were no two‐way interactions, but there was a three‐way condition × time × narcissism interaction.

**TABLE 2:** Analyses of children's winsorized muscle activity predicted by status loss versus gain condition, time, and children's narcissism levels

	Zygomaticus activity			Corrugator activity		
Intraclass correlation	0.81			0.95		
Fixed effects	*β*	*SE (β)*	*t*	*β*	*SE (β)*	*t*
Intercept	0.11	0.14	0.79	0.19	0.15	1.32
Condition	−0.23	0.20	−1.14	−0.38	0.21	−1.82
Time	−0.02	0.01	−4.33***	0.02	0.00	8.52***
Narcissism	−0.02	0.13	−0.12	0.13	0.14	0.93
Condition × time	0.01	0.01	1.08	0.03	0.00	7.71***
Condition × narcissism	0.07	0.20	0.35	−0.17	0.21	−0.79
Time × narcissism	0.01	0.00	0.43	0.01	0.00	3.79***
Condition × time × narcissism	0.03	0.01	3.77***	0.01	0.00	3.49***

3‐way interaction tests (time effect)	*β*	*SE (β)*	*t*	*β*	*SE (β)*	*t*
Low status						
Higher narcissism	ns	ns	ns	0.03	0.00	8.57***
Lower narcissism	ns	ns	ns	0.01	0.00	3.78***
High status						
Higher narcissism	0.02	0.01	2.11*	0.07	0.00	19.42***
Lower narcissism	−0.04	0.01	−5.56***	0.03	0.00	7.04***

Condition was dummy coded with “0” for Low Status and “1” for High Status. *p < 0.05 **p < 0.01 ***p < 0.001, *p* values calculated using Satterthwaite degrees of freedom.

**FIGURE 1:**
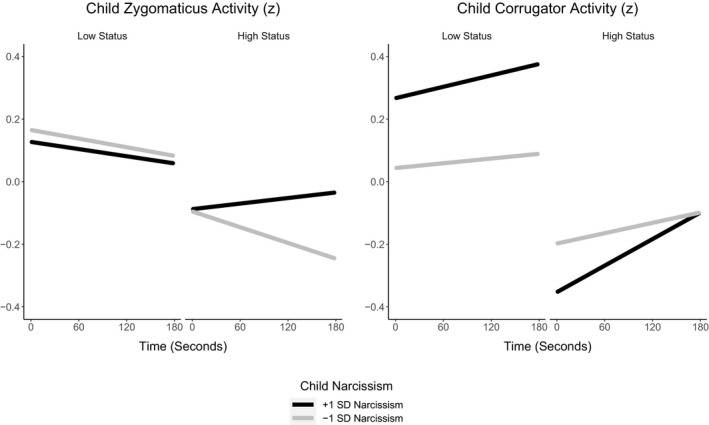
1 Conditional effects of time on children's zygomaticus (left panel) and corrugator (right panel) z‐transformed activity for values of children's narcissism at −1 *SD* (black lines) and +1 *SD* (yellow lines) in the low status (left half of panels) and the high‐status (right half of panels) conditions

We broke the three‐way interaction down by condition (Figure 1). In the low‐status condition, there was no time × narcissism interaction. In the high‐status condition, however, there was a two‐way time × narcissism interaction, *β* = 0.03, *SE* = 0.01, *t*(7,337) = 5.02, *p* < 0.001. Zygomaticus activity increased over time for children with higher (*M* + 1 *SD*) narcissism but decreased over time for children with lower (*M* − 1 *SD*) narcissism. Thus, during status gain, children with higher narcissism levels exhibited increasing zygomaticus activity, whereas those with lower narcissism levels exhibited decreasing zygomaticus activity.

#### 3.2.2 Corrugator activity

We tested the hypothesis that children's narcissism would be associated with increased corrugator activity when children lost status (Table 2). There were no effects of condition or narcissism, but there was an effect of time, with corrugator activity increasing over the course of the experiment. The two‐way interactions were qualified by a three‐way condition × time × narcissism interaction.

We broke the three‐way interaction down by condition (Figure 1). In the low‐status condition, there was a time × narcissism interaction, *β* = 0.01, *SE* < 0.01, *t*(7,516) = 3.77, *p* < 0.001. Corrugator activity increased more steeply for children with higher (*M* + 1 *SD*) narcissism than for children with lower (*M* – 1 *SD*) narcissism. In the high‐status condition, the time × narcissism interaction was similar but more pronounced, *β* = 0.02, *SE* < 0.01, *t*(7,337) = 7.78, *p* < 0.001. Corrugator activity increased more steeply over time for children with higher (*M* + 1 *SD*) narcissism than for children with lower (*M* – 1 *SD*) narcissism. Thus, children with higher narcissism levels exhibited increasing zygomaticus activity, especially during a status gain.

### 3.3 Parents' affective responses

Our second set of analyses examined parents' sensitivity to their children's status gains and losses.

#### 3.3.1 Zygomaticus activity, children's narcissism

We tested the hypothesis that children's narcissism would be associated with increased zygomaticus activity in parents when children gained status (Table 3). There were no effects of condition or children's narcissism, but there was an effect of time, with zygomaticus activity decreasing over time. There was also a time × condition interaction, which was qualified by a three‐way time × condition × children's narcissism interaction.

**TABLE 3:** Analyses of parents' winsorized muscle activity predicted by status loss versus gain condition, time, and children's narcissism levels

	Zygomaticus activity			Corrugator activity		
Intraclass correlation	0.97			0.93		
Fixed effects	*β*	*SE (β)*	*t*	*β*	*SE (β)*	*t*
Intercept	0.08	0.15	0.53	−0.02	0.15	−0.15
Condition	−0.16	0.22	−0.75	0.05	0.22	0.25
Time	−0.00	0.00	−2.56*	−0.03	0.00	−9.62***
Child narcissism	−0.05	0.14	−0.35	0.03	0.14	0.82
Condition × time	0.01	0.00	2.02*	0.05	0.00	12.57***
Condition × child narcissism	−0.04	0.22	−0.16	−0.09	0.22	−0.44
Time × child narcissism	−0.01	0.00	−4.66***	−0.00	0.00	−0.22
Condition × time × child narcissism	0.02	0.00	5.90***	−0.00	0.00	−1.08

3‐way interaction tests (time effect)	*β*	*SE (β)*	*t*	*β*	*SE (β)*	*t*
Low status						
Higher child narcissism	−0.01	0.00	−4.91***	ns	ns	ns
Lower child narcissism	0.00	0.00	1.34	ns	ns	ns
High status						
Higher child narcissism	0.01	0.00	3.12**	ns	ns	ns
Lower child narcissism	−0.01	0.00	−2.50*	ns	ns	ns

Condition was dummy coded with “0” for Low Status and “1” for High Status. *p < 0.05 **p < 0.01 ***p < 0.001, *p* values calculated using Satterthwaite degrees of freedom.

**FIGURE 2:**
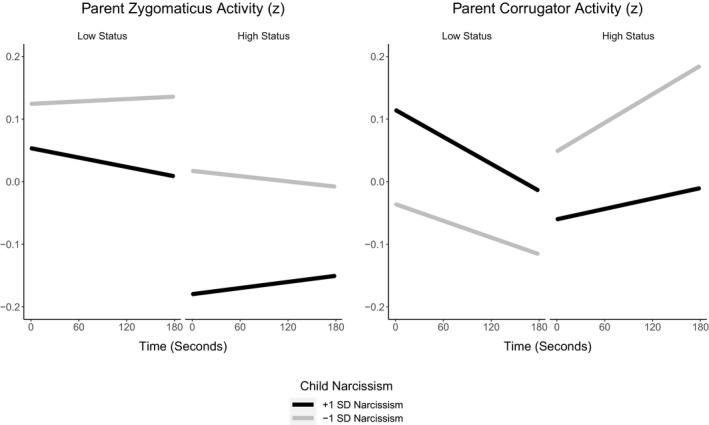
Conditional effects of time on parents' zygomaticus (left panel) and corrugator (right panel) z‐transformed activity for values of children's narcissism at −1 *SD* (black lines) and +1 *SD* (yellow lines) in the low status (left half of panels) and the high‐status (right half of panels) conditions

We broke the three‐way interaction down by condition (Figure 2). In the low‐status condition, there was a time × children's narcissism interaction, *β* = −0.01, *SE* < 0.01, *t*(7,516) = −4.12, *p* < 0.001. Zygomaticus activity decreased over time for parents of children with higher (*M* + 1 *SD*) narcissism but remained stable over time for parents of children with lower (*M* − 1 *SD*) narcissism. In the high‐status condition, there was also a time × children's narcissism interaction, *β* = 0.01, *SE* < 0.01, *t*(7,337) = 4.49, *p* < 0.001. Zygomaticus activity increased over time for parents of children with higher (*M* + 1 *SD*) narcissism but decreased over time for parents of children with lower (*M* − 1 *SD*) narcissism. Thus, during status loss, parents of children with higher narcissism levels exhibited decreasing zygomaticus activity, whereas, during status gain, they exhibited increasing zygomaticus activity.

#### 3.3.2 Corrugator activity, children's narcissism

We tested the hypothesis that children's narcissism would be associated with increased corrugator activity in parents when children lost status (Table 3). There were no effects of condition or children's narcissism. There was an effect of time, with corrugator activity decreasing over time. There was also a condition × time interaction, but no condition × time × children's narcissism interaction.

We broke the two‐way interaction down by testing the time effect per condition (Figure 2). In the low‐status condition, there was a significant decrease in corrugator activity over time, *β* = −0.03, *SE* < 0.01, *t*(7,517) = −7.55, *p* < 0.001. In the high‐status condition, however, there was a significant increase in corrugator activity over time, *β* = 0.02, *SE* < 0.01, *t*(7,338) = 13.47, *p* < 0.001. Thus, during status loss, parents exhibited decreasing corrugator activity, whereas, during status gain, parents exhibited increasing corrugator activity.

#### 3.3.3 Zygomaticus activity, parental overvaluation

We tested the hypothesis that parental overvaluation would be associated with increased zygomaticus activity in parents when children gained status (Table 4). There were no effects of condition or parental overvaluation. There was an effect of time, with zygomaticus activity decreasing over time. There was also a condition × time × parental overvaluation interaction.

**TABLE 4:** Analyses of parents' winsorized muscle activity predicted by status loss versus gain condition, time, and parental overvaluation levels

	Zygomaticus activity			Corrugator activity		
Intraclass correlation	0.97			0.93		
Fixed effects	*β*	*SE (β)*	*t*	*β*	*SE (β)*	*t*
Intercept	0.08	0.15	0.54	−0.01	0.15	−0.08
Condition	−0.15	0.21	−0.67	0.05	0.22	0.25
Time	−0.00	0.00	−1.97*	−0.03	0.00	−9.89***
Overvaluation	−0.00	0.16	−0.03	0.06	0.16	0.40
Condition × time	0.00	0.00	0.69	0.06	0.00	13.16***
Condition × overvaluation	−0.10	0.22	−0.48	−0.14	0.22	−0.64
Time × overvaluation	0.00	0.00	1.38	−0.01	0.00	−2.25*
Condition × time × overvaluation	0.01	0.00	4.57***	−0.01	0.00	−1.26

3‐way interaction tests (time effect)	*β*	*SE (β)*	*t*	*β*	*SE (β)*	*t*
Low status						
Higher overvaluation	ns	ns	ns	ns	ns	ns
Lower overvaluation	ns	ns	ns	ns	ns	ns
High status						
Higher overvaluation	0.01	0.00	−5.82***	ns	ns	ns
Lower overvaluation	−0.02	0.00	5.50***	ns	ns	ns

Condition was dummy coded with “0” for Low Status and “1” for High Status. *p < 0.05 **p < 0.01 ***p < 0.001, *p* values calculated using Satterthwaite degrees of freedom.

**FIGURE 3:**
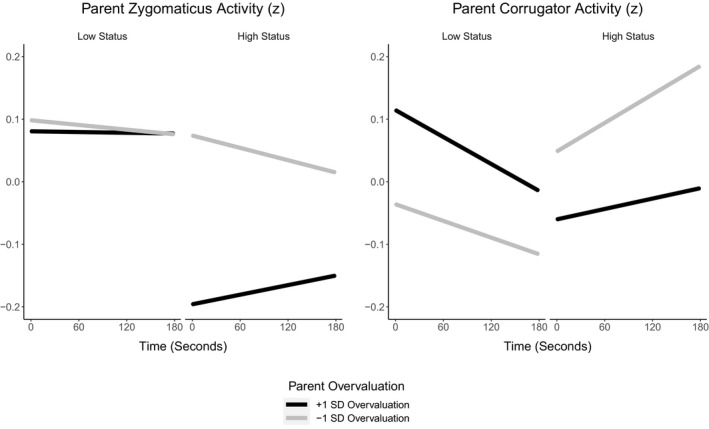
Conditional effects of time on parents' zygomaticus (left panel) and corrugator (right panel) z‐transformed activity for values of parental overvaluation at −1 *SD* (black lines) and +1 *SD* (yellow lines) in the low status (left half of panels) and the high‐status (right half of panels) conditions

We broke the three‐way interaction down by condition (Figure 3). In the low‐status condition, there was no time × parental overvaluation interaction. In the high‐status condition, however, there was a time × parental overvaluation interaction, *β* = 0.02, *SE* < 0.01, *t*(7,337) = 9.64, *p* < 0.001. Zygomaticus activity increased over time for more (*M* + 1 *SD*) overvaluing parents but decreased over time for less (*M* – 1 *SD*) overvaluing parents. Thus, during status gain, more overvaluing parents exhibited increasing zygomaticus activity.

#### 3.3.4 Corrugator activity, parental overvaluation

We tested the hypothesis that parental overvaluation would be associated with increased corrugator activity in parents when children lost status (Table 4). There were no effects of condition or parental overvaluation. However, there was an effect of time, with corrugator activity decreasing over time. There was also a time × condition, as well as a time × overvaluation interaction. There was no condition × time × parental overvaluation interaction.

First, we broke down the time × condition interaction by testing the time effect per condition (Figure 3). In the low‐status condition, corrugator activity decreased over time, *β* = −0.03, *SE* < 0.01, *t*(7,517) = −7.55, *p* < 0.001. In the high‐status condition, however, corrugator activity increased over time, *β *= 0.02, *SE* < 0.01, *t*(7,338) = 13.47, *p* < 0.001. Thus, during status loss, parents exhibited decreasing corrugator activity, whereas during status gain, parents exhibited increasing corrugator activity.

Second, we broke down the time × parental overvaluation interaction by testing the time effect for different parental overvaluation levels (Figure 3). Corrugator activity decreased over time for more (*M* + 1 *SD*) overvaluing parents, *β *= −0.01, *SE* < 0.01, *p* < 0.001, but increased over time for less (*M* – 1 *SD*) overvaluing parents, *β* = 0.01, *SE* < 0.01, *p* < 0.001. Thus, during both status gain and loss, more overvaluing parents exhibited decreasing corrugator activity.

### 3.4 Auxiliary analyses

Auxiliary analyses are presented in the Supplementary Material.

#### 3.4.1 Robustness tests

We repeated main analyses excluding observations above 2 *SD* of z‐standardized winsorized muscle activity. Findings were the same, except for corrugator activity in parents: During status loss, parents of children with higher narcissism levels and more overvaluing parents exhibited increasing corrugator activity.

#### 3.4.2 Specificity tests

We compared results for narcissism to those for self‐esteem. Self‐esteem also involves favorable self‐views (Brummelman et al., 2016) but, unlike narcissism, protects against psychopathology (Hyatt et al., 2018). Before the task, children completed the Lifespan Self‐Esteem Scale (Harris et al., 2018). First, we repeated the main analyses in children, while controlling for self‐esteem. The pattern of results for narcissism did not change. Second, we conducted the same analyses for self‐esteem that we conducted for narcissism. Like narcissism, self‐esteem was associated with increasing zygomaticus and corrugator activity over time when children gained status. Unlike narcissism, self‐esteem was associated with decreasing corrugator activity over time when children lost status. Thus, the overall pattern of results was specific to narcissism.

## 4 DISCUSSION

This randomized experiment used fEMG to examine, for the first time, whether childhood narcissism is related to status sensitivity, and whether this sensitivity is shared with parents. Positive and negative affect were indexed via zygomaticus and corrugator activity. Children with higher narcissism levels experienced status loss more negatively, with steeper increases in negative affect. They also experienced status gain with increases in both positive and negative affect. Their parents mirrored these affective responses, although they experienced status gain as unambiguously positive, with steeper increases only in positive affect. Findings were modest in size yet robust.

### 4.1 Theoretical implications

Children with higher narcissism levels exhibited intensified affective responses to status gains and losses. This provides the first direct evidence that childhood narcissism is characterized by heightened status sensitivity, which could indicate an underlying affective‐motivational system geared toward status (Brummelman & Sedikides, 2020; Grapsas et al., 2020; Morf & Rhodewalt, 2001; Zeigler‐Hill et al., 2018). Parents of children with higher narcissism levels showed affective responses similar to their children's, which suggests that status sensitivity can be intergenerationally transmitted. These results were distinct from those for self‐esteem. Thus, our work shows, for the first time, that the affective signatures of narcissism emerge in childhood, are shared with parents, and are unique to narcissism. More broadly, our work concurs with the notion that personality traits, such as narcissism, reflect unique affective signatures (Dufner et al., 2015; McClelland, 1987; Mischel & Shoda, 1995). Our findings suggest that in children with higher narcissism levels, status gains trigger both positive and negative affect. Supplementary analyses show that for them, positive and negative affect during status gain did not co‐occur in time (Supplementary Material, pp. 52–53). This indicates that these children did not experience status gain as more ambivalent (i.e., simultaneously positive and negative); rather, they cycled between positive and negative affect.

Why might gaining status trigger negative affect in children with high narcissism levels? In competitive settings, status gains might seem fleeting, raising concerns about losing status—especially so in children with a stronger status motive, such as children with higher narcissism levels (Case et al., 2020; Kakkar et al., 2019). These negative‐affective states might have adaptive value, preparing children to protect or even enhance their status (Case et al., 2020; Huang et al., 2017).

Parents of children with higher narcissism levels and more overvaluing parents showed intensified affective responses to their child's status gains and losses. Why might such an intergenerational overlap in status sensitivity exist? Given that narcissism is partly heritable (Vernon et al., 2008), children with high narcissism levels might inherit their parents' status sensitivity. Additionally, parents might transmit status sensitivity through socialization (Brummelman, 2018; Brummelman & Sedikides, 2020). For example, parents might visibly show their pleasure and displeasure in their child's status gains and losses via their facial expressions. Consistent with work on social referencing (Bos et al., 2016) and reinforcement learning (Berridge, 2000), children might detect parents' affective reactions and interpret them as signs that they should pursue status to please them. However, an alternative explanation might be that parents accurately inferred and mirrored via their affective responses to how their child would feel. Future work should identify the sources of this intergenerational overlap.

### 4.2 Strengths, limitations, and future research

Strengths of our study include its developmental timing, ecologically valid social media task, and experimental design with real‐time continuous physiological tracking of affect. Our study also has limitations. First, we manipulated status gains and losses in a one‐shot social media interaction. Future research should examine how children respond to social media feedback and other experiences of status gain and loss over days, weeks, or months, as these experiences might have cumulative effects (Lee et al., 2020). Second, the task was designed to examine affective responses to the overall experience of status gain or loss over time, rather than to individual, isolated likes. Future research should examine how children respond to individual likes (Sherman et al., 2016). Third, we used fEMG to index affect. Although fEMG reliably assesses affect (Barrett et al., 2019), we do not claim a one‐to‐one mapping of facial muscle activity to affect, and we cannot rule out the possibility that facial muscle activity might reflect more than affect (e.g., corrugator activity can indicate mental effort; Kraaijenvanger et al., 2017).

## 5 CONCLUSION

Children's lives are rife with experiences of status gains and losses. Our findings show that the affective consequences of these experiences are especially pronounced in children with high narcissism levels and their parents. These findings suggest that an affective‐motivational system may underlie childhood narcissism and pave the way to research how this system becomes intergenerationally transmitted.

## CONFLICT OF INTEREST

The authors declared that there were no conflicts of interest with respect to the authorship or the publication of this article.

## Supporting information

Supplementary Material

## Data Availability

Data available on request from the authors.
