# Supplementary material for: Climbing up or falling down: Narcissism predicts physiological sensitivity to social status in children and their parents
Source: Dev Sci. 2020 Nov 25;24(4):e13062. doi: 10.1111/desc.13062 (PMC8365742; doi:10.1111/desc.13062)
Supplement: Supplementary file 1 — Supplementary Material [file DESC-24-e13062-s001.docx]

**Method Supplement**

**Social Media task**

The social media task was presented as a competitive popularity game (Figure S1) in an online social media platform that participants were verbally informed was ostensibly under development. Children first created a personal profile by entering their initials (Figure S2), selecting a personal avatar from a list of predesigned portraits of youths (Figure S4), and writing a paragraph about themselves (Figure S5). Children viewed the game instructions, then the visual layout of the game. In the high-status and the low-status condition, children were informed that contestants would read one another’s profiles and give likes (similar to Facebook likes) to any contestant, aside from themselves, they found special and exceptional. A notification popped up each time any contestant received a like. Likes were visible below each contestant’s profile. Each contestant’s position in the popularity hierarchy was shown in a ranking board, with the child at the top of the board being the contestant with most likes at that time (Figures S6, S7). Children in the no likes condition (intended to be neutral) were not informed about giving or gaining likes (Figure S8), and the visual layout of the game excluded the likes below the profiles, the notifications, and the ranking board (Figure S9).

In the high-status condition, children soon started sharing the first position with fictitious peers (S10). Over time, they shared it with fewer and fewer (S11) and finished the task second in ranking (S12).

In the low-status condition, children soon dropped to one of the lowest ranking positions (S13), and short thereafter to the lowest ranking (S14), remaining there for the task duration (S15).

In the no likes condition, children did not interact with fictitious peers (S16).


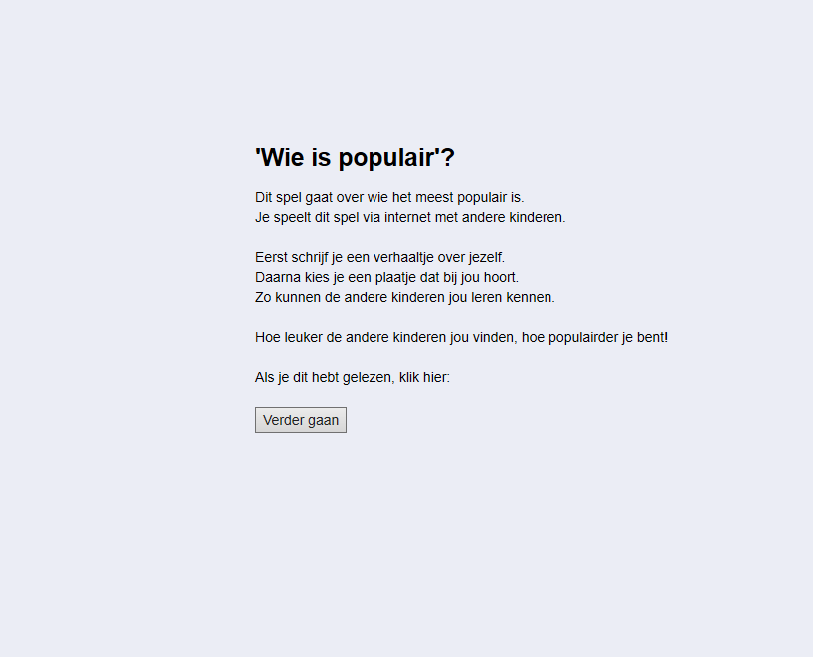


*Figure S1.* All conditions, page 1 of social media task. Introduction to the game.


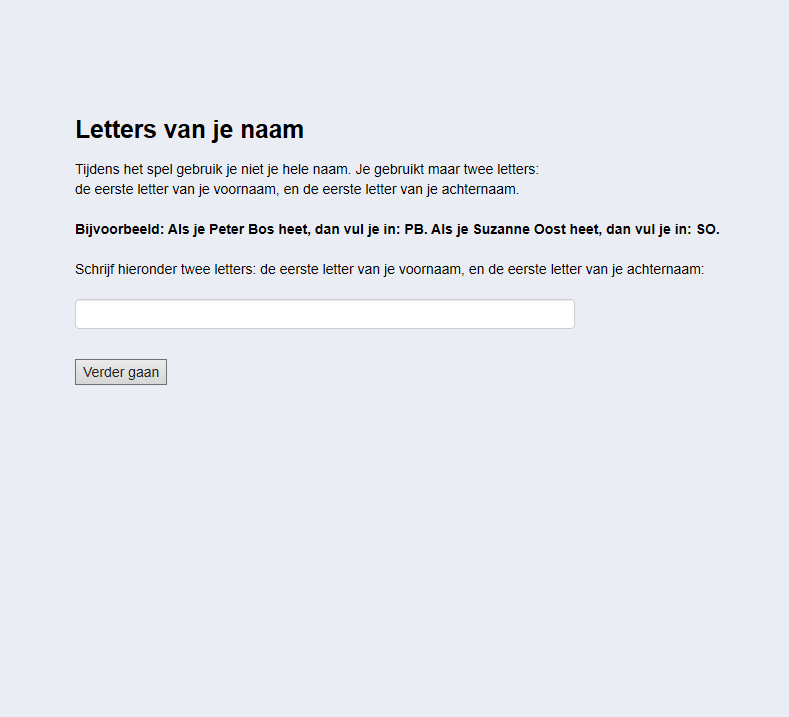


*Figure S2.* All conditions, page 2 of social media task. Entering personal initials.

*
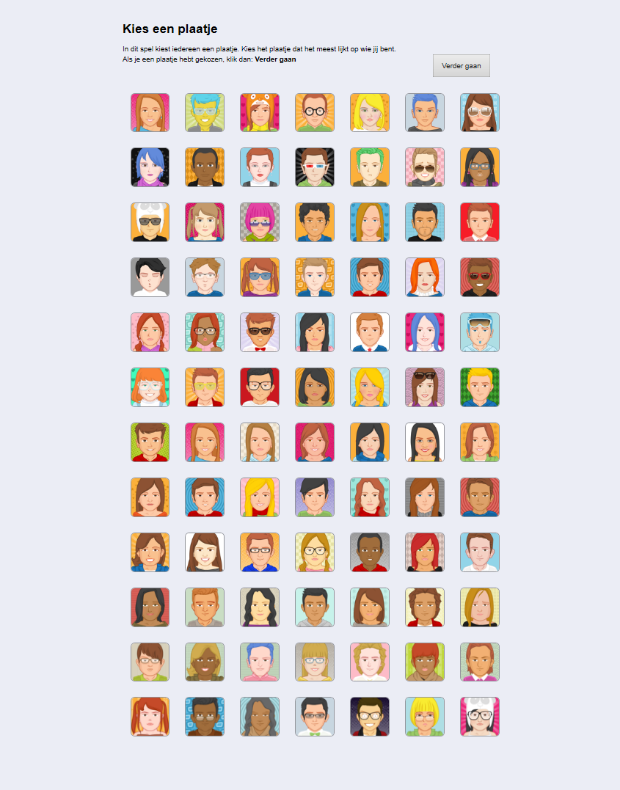
*

*Figure S4.* All conditions, page 3 of social media task. Selecting a personal avatar.


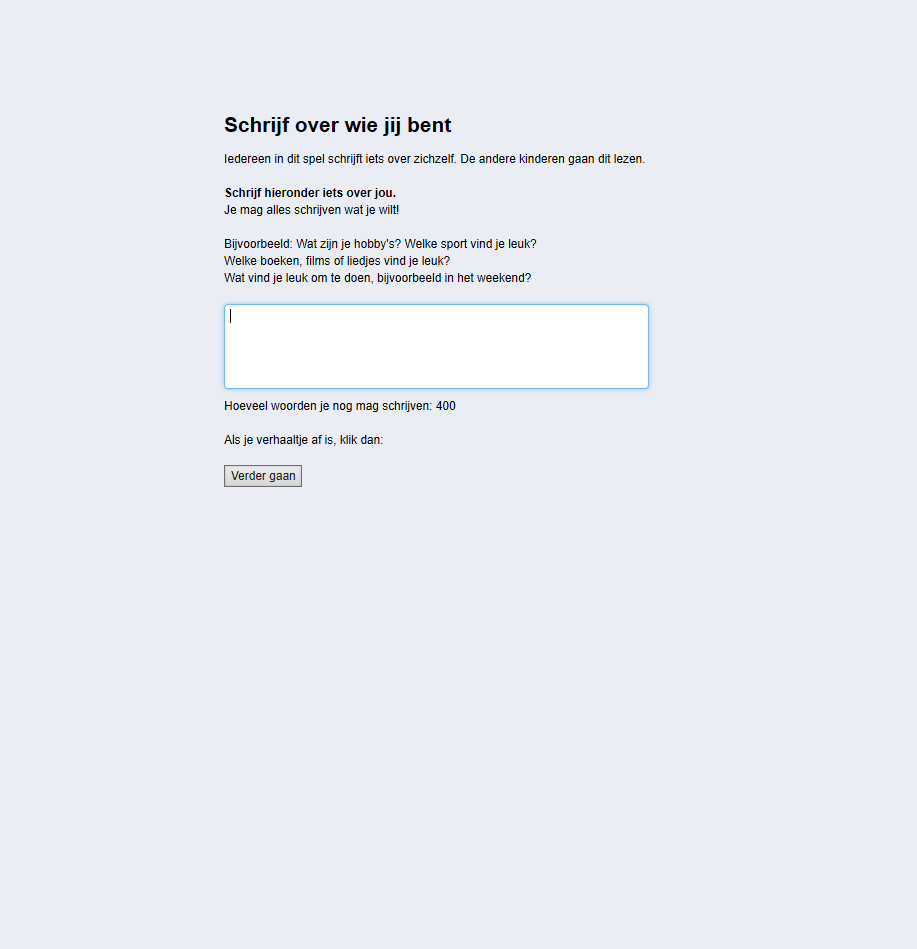


*Figure S5.* All conditions, page 4 of social media task. Writing a self-description


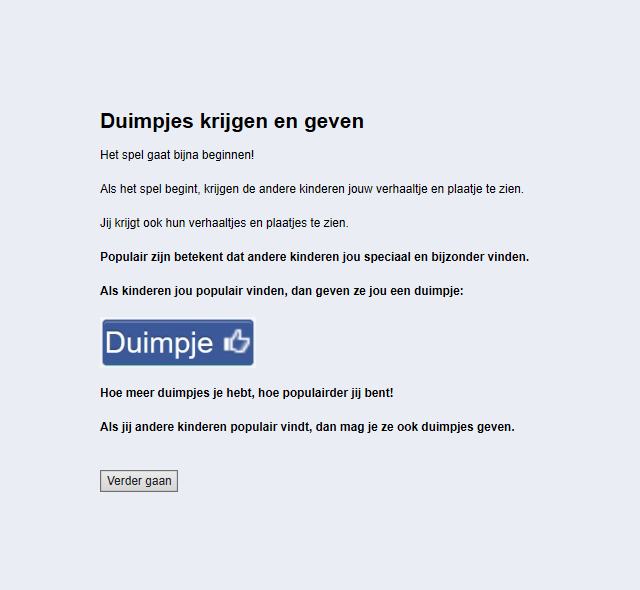


*Figure S6.* Low- and high-status conditions, page 5 of social media task. Instructions about likes. In the game, likes are given to those who are deemed as popular, that is, special and exceptional.


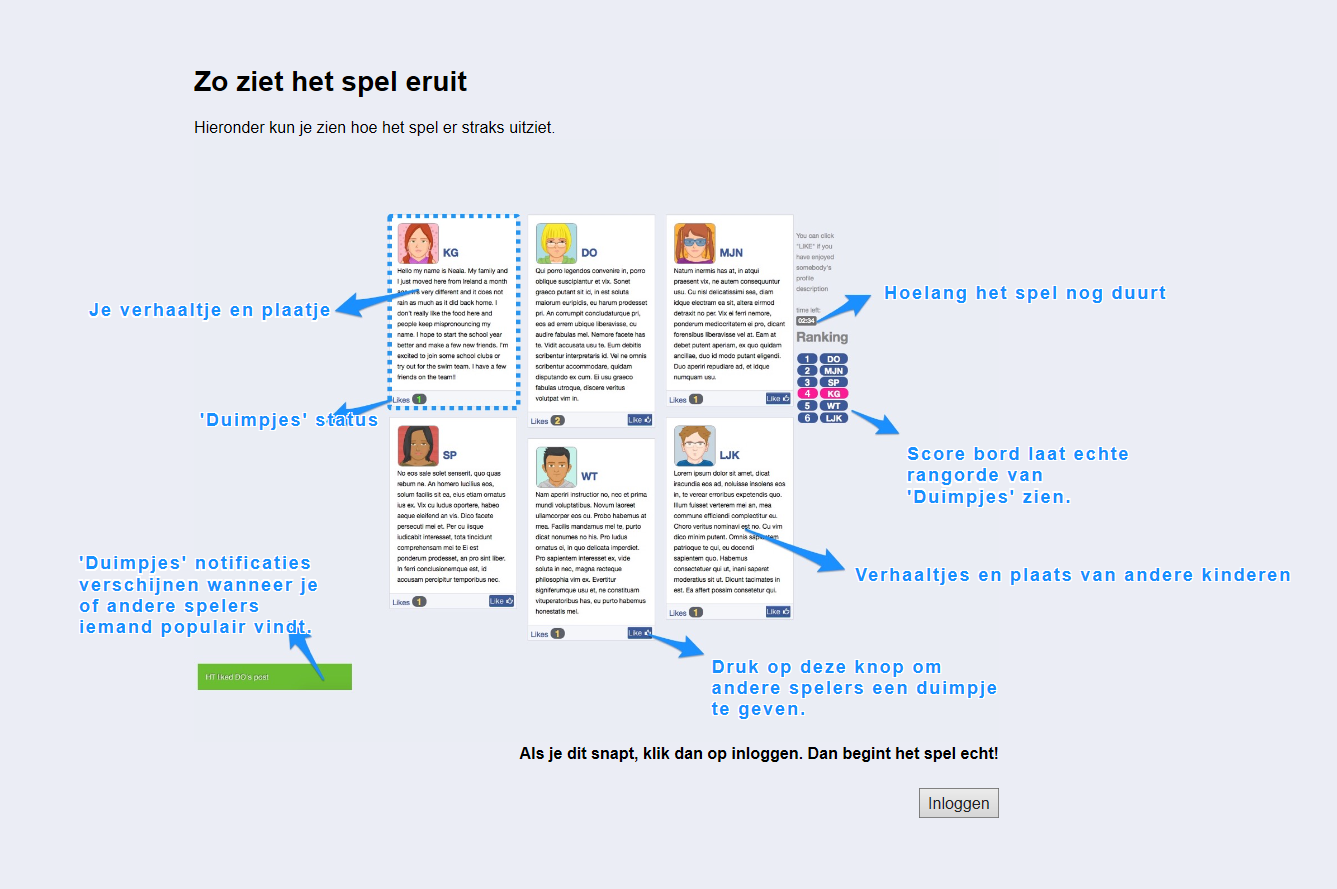


*Figure S7.* Low- and high-status conditions, page 6 of social media task. Visual layout of the game.


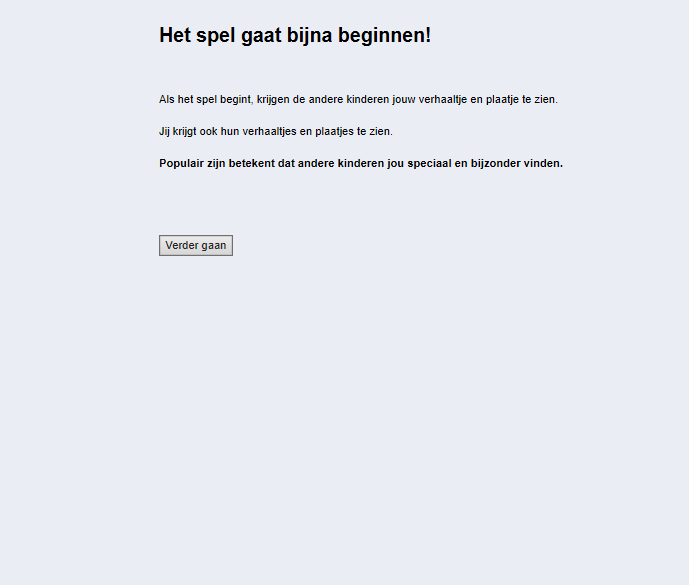


*Figure S8.* No likes condition, page 5 of social media task.


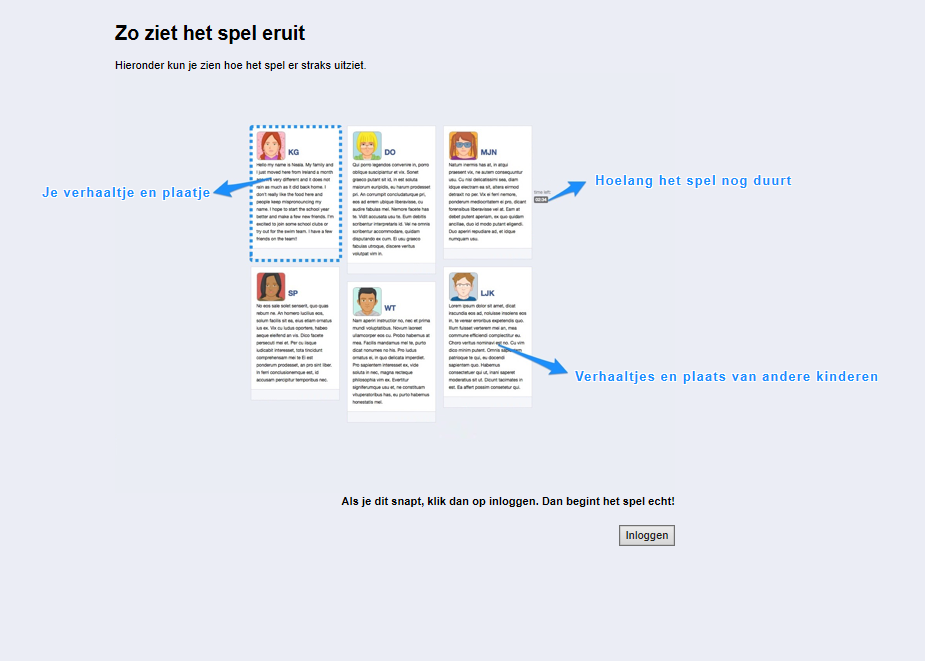


*Figure S9.* No likes condition, page 6 of social media task. Visual layout of the game.


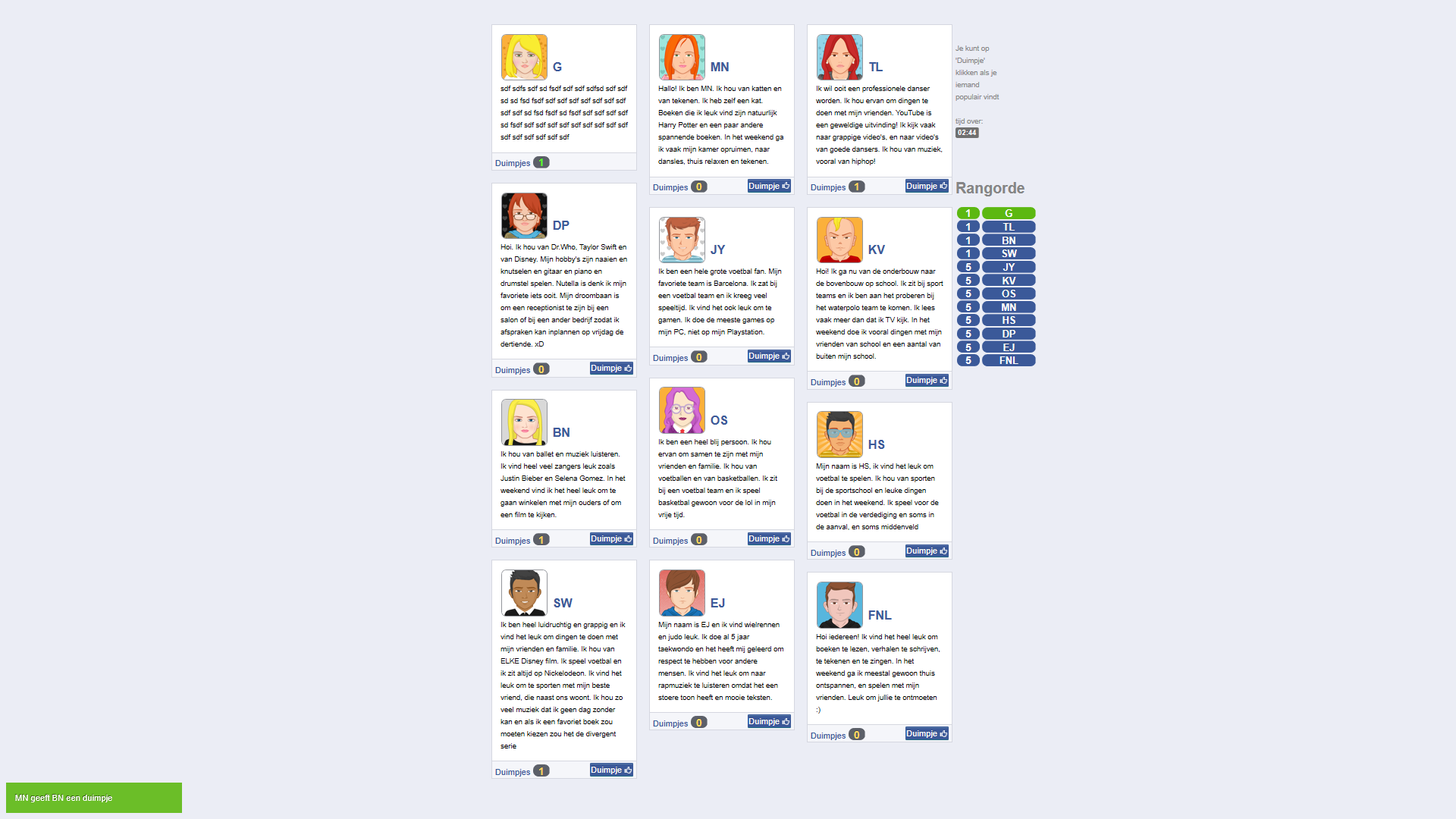


Figure *S10*. High status condition. Initial sharing of first position with other peers.


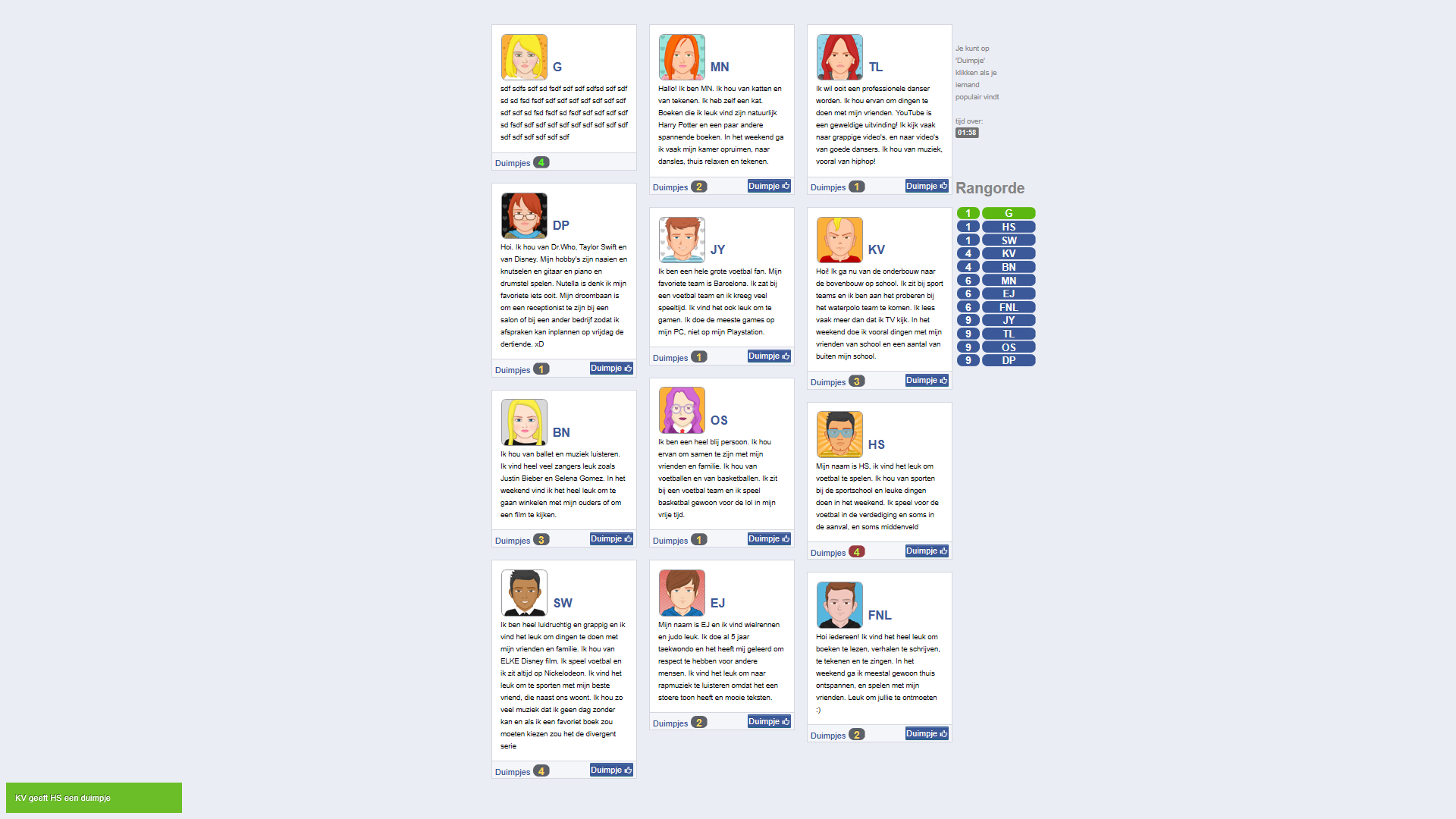


Figure *S11.* High status condition. Sharing first position with fewer peers.


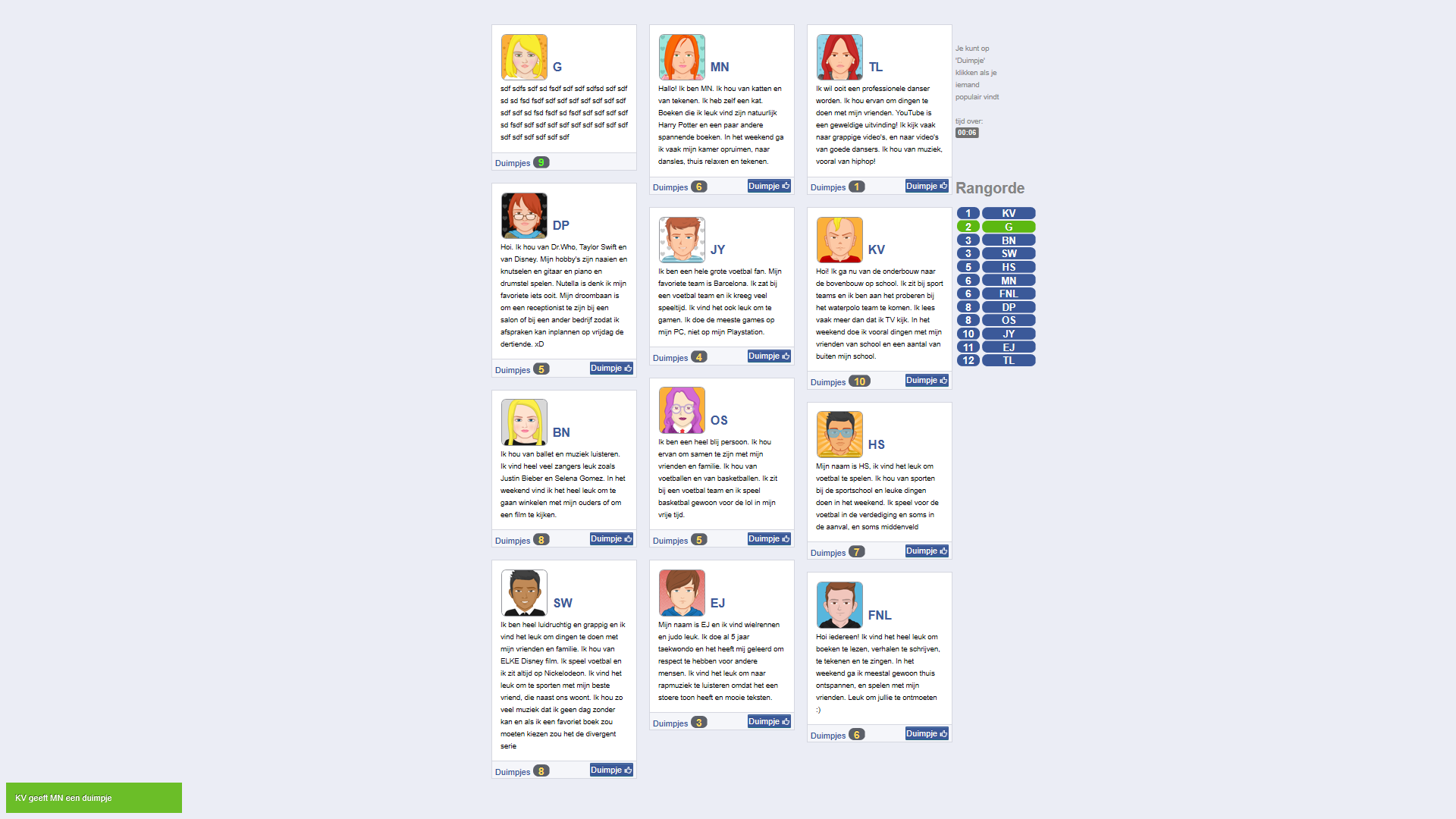


Figure *S12.* High status condition. Approaching the end of the game as second in ranking.


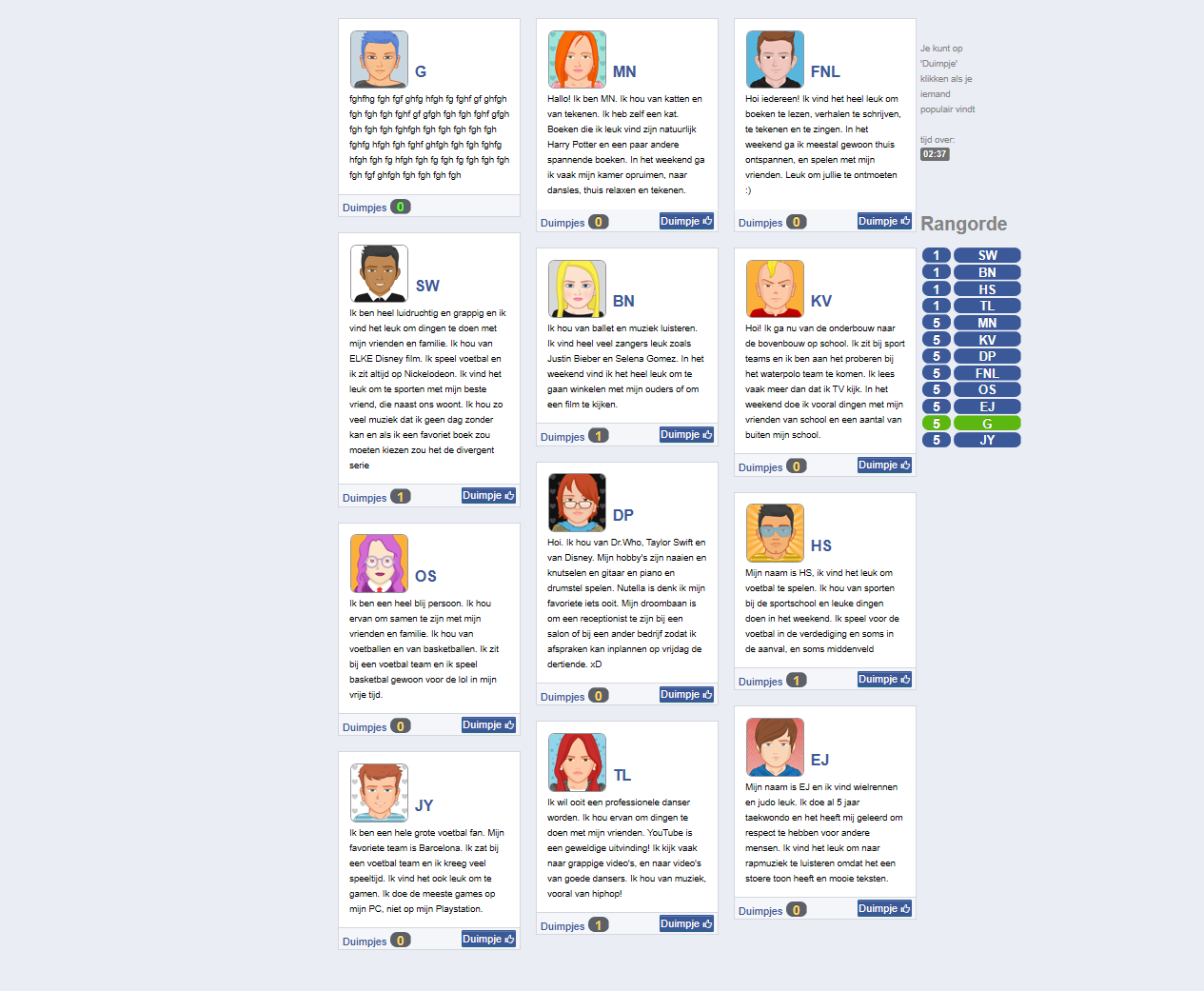


Figure *S13*. Low status condition. Initial drop in ranking.


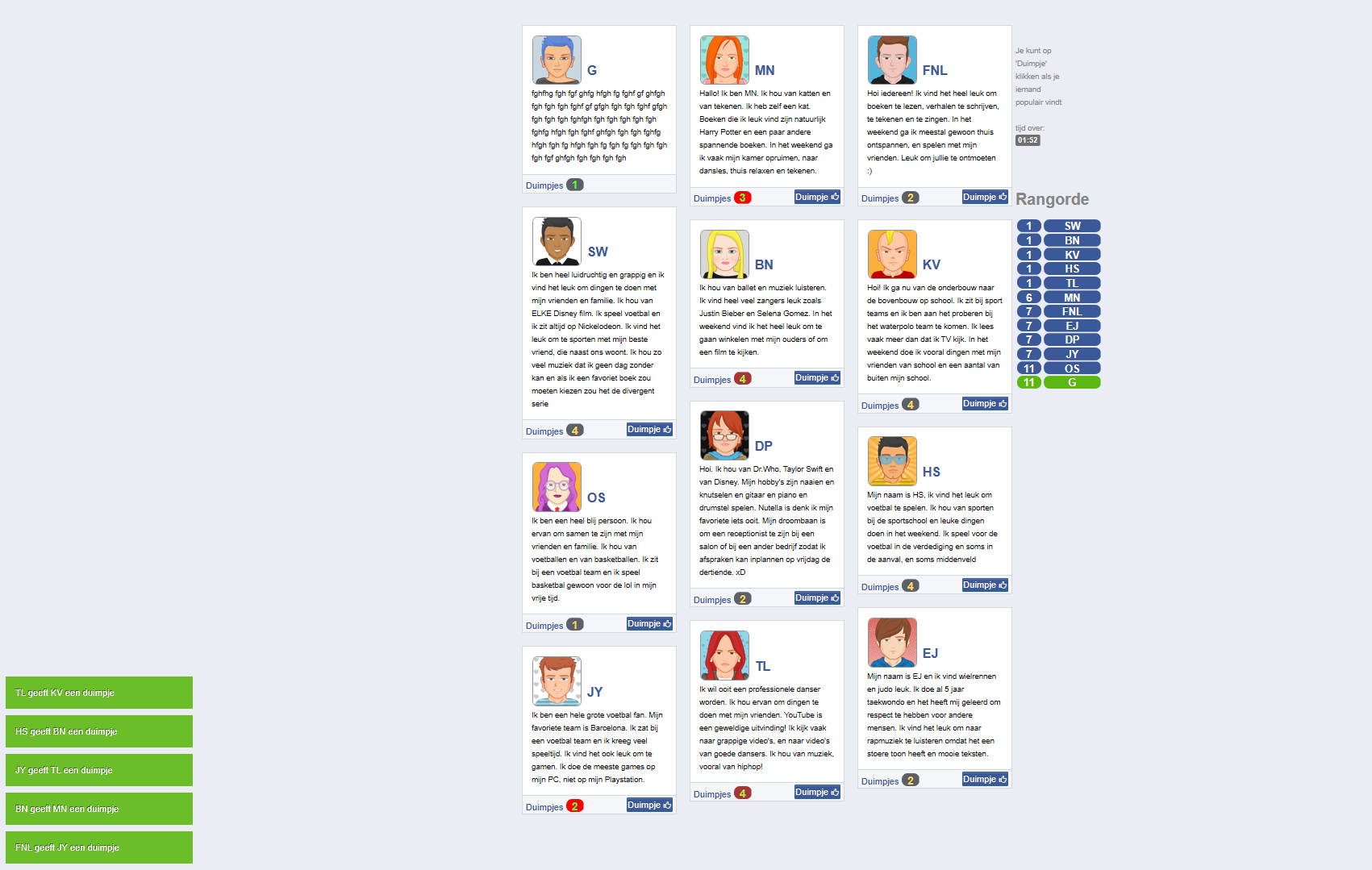


Figure *S14.* Low status condition. Participant in the bottom of ranking.


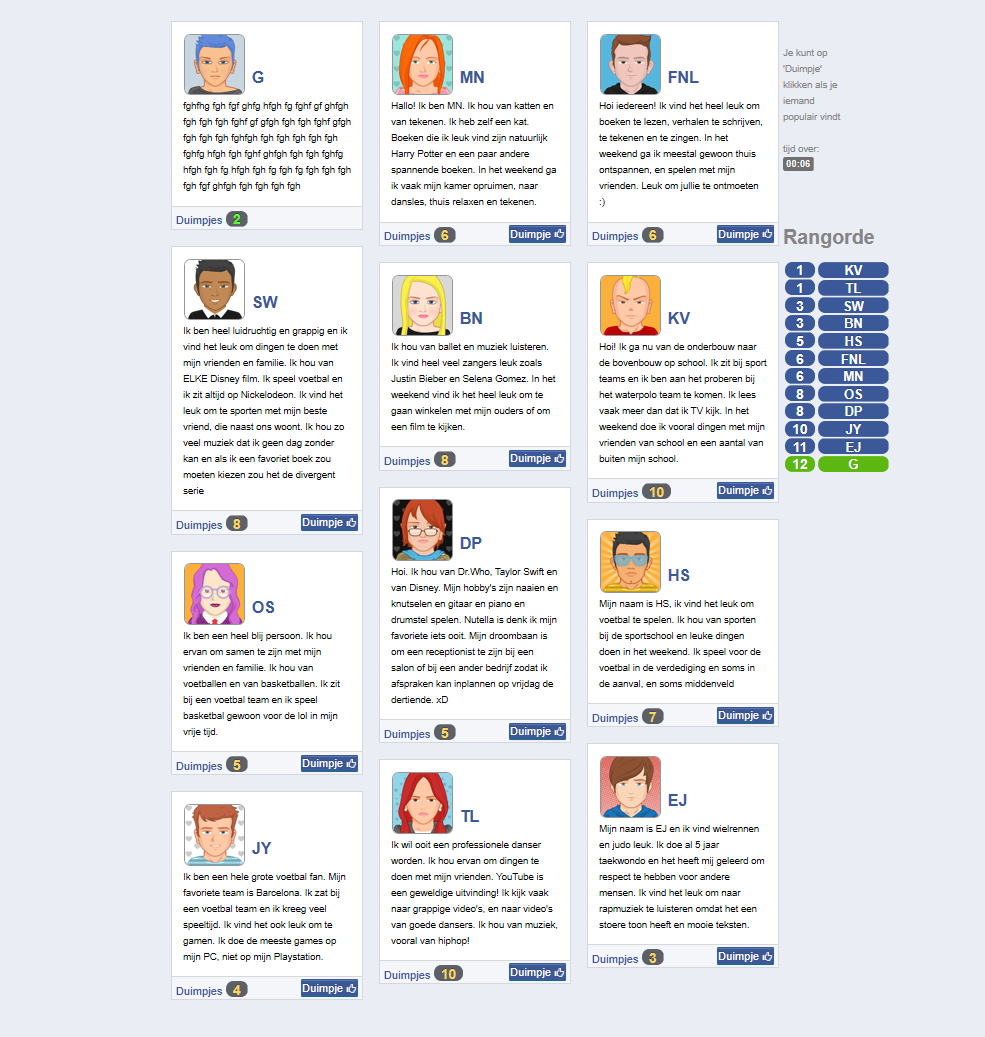


Figure *S15.* Low status condition. Approaching the end of the game as last in ranking.


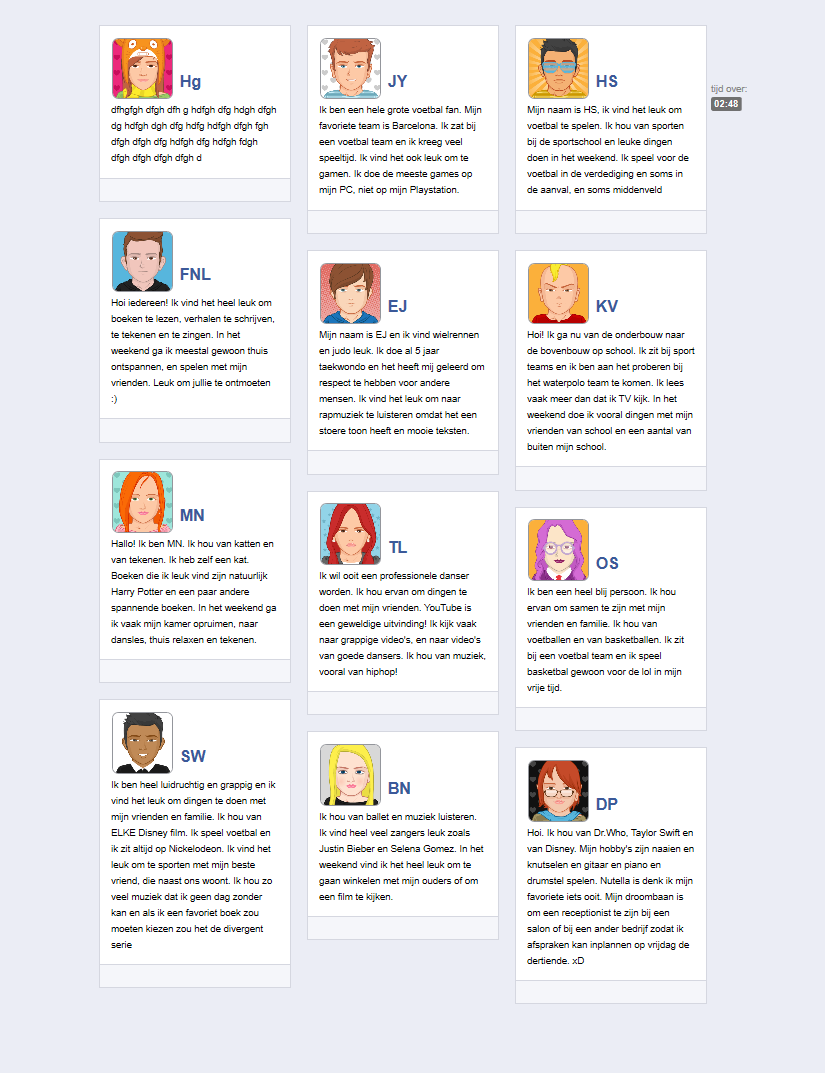


Figure *S16*. No likes condition. No interaction during the game.

**EMG Recordings**

Facial EMG equipment was placed on children and parents prior to questionnaire completion and remained attached throughout the testing session. Following standard procedures (Fridlund & Cacioppo, 1986), we first cleaned participants’ skin using alcohol pads. We then placed two bipolar Ag/AgCl electrodes 15 mm from one another on participants’ right zygomaticus major muscle, and two bipolar Ag/AgCl electrodes 15 mm from one another on participants’ right corrugator muscle. We also placed a common reference electrode on the forehead. Electrodes had a 2mm diameter contact area.

EMG recordings were retrieved at a sampling rate of 2000 Hz through a Biopac MP150 data acquisition unit. Signals were filtered online (10 Hz highpass filter, 500Hz lowpass filter) and offline (30 Hz – 500 Hz bandpass filter to remove movement artifacts and prevent aliasing, 50 Hz notch filter to remove power line interference; van Boxtel, 2010). Filtered signals were rectified and aggregated per second.

**Power Analyses**

We ran post-hoc power analyses on the effect sizes extracted from each highest-order interaction included in the main analyses. For each effect, 1000 Monte Carlo simulations for a = 0.05 were run. Overall, power analyses indicated that the current sample size yielded sufficient power to detect the effect sizes that were found (Table S1).

Table S1

*Post Hoc Power Analyses for Effect Sizes from Main Analyses*

| Analysis | Highest-Order Interaction | Observed Power |
| --- | --- | --- |
| Children, zygomaticus activity | Condition*Time*Children’s Narcissism | 96.00% |
| Children, corrugator activity | Condition*Time*Children’s Narcissism | 93.70% |
| Parents, zygomaticus activity, Children’s Narcissism | Condition*Time*Children’s Narcissism | 100% |
| Parents, corrugator activity, Children’s Narcissism | Condition*Time | 100% |
| Parents, zygomaticus activity, Parental Overvaluation | Condition*Time*Children’s Narcissism | 99.10% |
| Parents, corrugator activity, Parental Overvaluation | Condition*Time | 100% |
| Parents, corrugator activity, Parental Overvaluation | Time*Parental Overvaluation | 63.20% |

**Preliminary Analyses**

Random assignment was successful. There were no condition differences in children’s age, *t*(81) = -0.53, *p* = .595, narcissism, *t*(81) = 0.59, *p* = .556, or sex/gender, *χ*^2^(1) = 0.11, *p* = .741, nor in parents’ age, *t*(81) = 1.05, *p* = .297, parental overvaluation, *t*(81) = 1.8, *p* = 0.08, or parent’s sex/gender, *χ*^2^(1) = 0.29, *p* = .589. Children’s and parents’ baseline muscle activity did not differ by condition, children’s narcissism levels, or parental overvaluation levels.

**Model Comparison**

In main analyses, we computed p-values using the lmerTest package version 3.1.1 (Kuznetsova, Brockhoff, & Christensen, 2017). In Table S2 below, we present an alternative method of p-value computation, via model comparison. This approach yielded the same results.

Table S2

*P-Values of Main Model Effects Estimated via Model Comparison*

| **Zygomaticus Activity** | | | | | | |  | | **Corrugator Activity Models** | | | | | | | |  | |
| --- | --- | --- | --- | --- | --- | --- | --- | --- | --- | --- | --- | --- | --- | --- | --- | --- | --- | --- |
| **Fixed Effects** | **Df** | **AIC** | **BIC** | **LL** | **-2LL** | ***χ^2^*** | **Sig.** |  | | **Df** | **AIC** | **BIC** | **LL** | **-2LL** | ***χ^2^*** | **Sig.** | |  |
| **Children, Child Narcissism Models** | | | | | | | | | | | | | | | | |  | |
| Null Model | 3 | 18336 | 18359 | -9165 | 18330 | - | - |  | | 3 | -652.20 | -629.30 | 329.10 | -658.20 | - | - | |  |
| **Time** | 4 | 18315 | 18346 | -9154 | 18307 | 22.80 | 0.000 |  | | 4 | -1036.30 | -1005.80 | 522.10 | -1044.30 | 386.13 | 0.000 | |  |
| Time, **Condition** | 5 | 18316 | 18354 | -9153 | 18306 | 1.27 | 0.260 |  | | 5 | -1037.40 | -999.40 | 523.70 | -1047.40 | 3.15 | 0.076 | |  |
| Time, Condition, **Narcissism** | 6 | 18318 | 18364 | -9153 | 18306 | 0.02 | 0.891 |  | | 6 | -1035.70 | -990.10 | 523.90 | -1047.70 | 0.31 | 0.577 | |  |
| Time, Condition, Narcissism,  **Time*Condition** | 7 | 18318 | 18372 | -9152 | 18304 | 1.88 | 0.170 |  | | 7 | -1102.00 | -1048.70 | 558.00 | -1116.00 | 68.24 | 0.000 | |  |
| Time, Condition, Narcissism,  Time*Condition, **Time*Narcissism**, | 8 | 18306 | 18367 | -9145 | 18290 | 13.89 | 0.000 |  | | 8 | -1161.90 | -1101.00 | 588.90 | -1177.90 | 61.92 | 0.000 | |  |
| Time, Condition, Narcissism,  Time*Condition, Time*Narcissism, **Condition*Narcissism** | 9 | 18308 | 18377 | -9145 | 18290 | 0.12 | 0.725 |  | | 9 | -1160.50 | -1092.00 | 589.30 | -1178.50 | 0.61 | 0.433 | |  |
| Time, Condition, Narcissism,  Time*Condition, Time*Narcissism, Condition*Narcissism, **Time*Condition*Narcissism** | 10 | 18296 | 18372 | -9138 | 18276 | 14.19 | 0.000 |  | | 10 | -1170.70 | -1094.60 | 595.30 | -1190.70 | 12.18 | 0.000 | |  |
| **Parents, Child Narcissism Models** | | | | | | | | | | | | | | | | | | |
| Null Model | 3 | -11035 | -11012 | 5521 | -11041 | - | - |  | | 3 | 3006 | 3029 | -1500 | 3000 | - | - | |  |
| **Time** | 4 | -11035 | -11004 | 5521 | -11043 | 1.47 | 0.226 |  | | 4 | 3007 | 3037 | -1499 | 2999 | 1.36 | 0.243 | |  |
| Time, **Condition** | 5 | -11033 | -10995 | 5522 | -11043 | 0.62 | 0.433 |  | | 5 | 3008 | 3047 | -1499 | 2998 | 0.06 | 0.812 | |  |
| Time, Condition, **Narcissism** | 6 | -11031 | -10986 | 5522 | -11043 | 0.35 | 0.553 |  | | 6 | 3010 | 3056 | -1499 | 2998 | 0.01 | 0.937 | |  |
| Time, Condition, Narcissism,  **Time*Condition** | 7 | -11034 | -10980 | 5524 | -11048 | 4.09 | 0.043 |  | | 7 | 2857 | 2910 | -1421 | 2843 | 155.62 | 0.000 | |  |
| Time, Condition, Narcissism,  Time*Condition, **Time*Narcissism**, | 8 | -11033 | -10972 | 5524 | -11049 | 1.28 | 0.259 |  | | 8 | 2857 | 2918 | -1421 | 2841 | 1.42 | 0.234 | |  |
| Time, Condition, Narcissism,  Time*Condition, Time*Narcissism, **Condition*Narcissism** | 9 | -11031 | -10962 | 5524 | -11049 | 0.03 | 0.871 |  | | 9 | 2859 | 2928 | -1421 | 2841 | 0.19 | 0.660 | |  |
| Time, Condition, Narcissism,  Time*Condition, Time*Narcissism, Condition*Narcissism, **Time*Condition*Narcissism** | 10 | -11064 | -10988 | 5542 | -11084 | 34.79 | 0.000 |  | | 10 | 2860 | 2936 | -1420 | 2840 | 1.16 | 0.282 | |  |
| **Parents, Parental Overvaluation Models** | | | | | | | | | | | | | | | | | |  |
| Null Model | 3 | -11035 | -11012 | 5521 | -11041 | - | - |  | | 3 | 3006 | 3029 | -1500 | 3000 | - | - | |  |
| **Time** | 4 | -11035 | -11004 | 5521 | -11043 | 1.47 | 0.226 |  | | 4 | 3007 | 3037 | -1499 | 2999 | 1.36 | 0.243 | |  |
| Time, **Condition** | 5 | -11033 | -10995 | 5522 | -11043 | 0.62 | 0.433 |  | | 5 | 3008 | 3047 | -1499 | 2998 | 0.06 | 0.812 | |  |
| Time, Condition, **Overvaluation** | 6 | -11031 | -10986 | 5522 | -11043 | 0.29 | 0.589 |  | | 6 | 3010 | 3056 | -1499 | 2998 | 0.01 | 0.924 | |  |
| Time, Condition, Narcissism,  **Time*Condition** | 7 | -11034 | -10980 | 5524 | -11048 | 4.09 | 0.043 |  | | 7 | 2857 | 2910 | -1421 | 2843 | 155.62 | 0.000 | |  |
| Time, Condition, Overvaluation,  Time*Condition, **Time*Overvaluation**, | 8 | -11078 | -11017 | 5547 | -11094 | 46.69 | 0.000 |  | | 8 | 2838 | 2899 | -1411 | 2822 | 21.09 | 0.000 | |  |
| Time, Condition, Overvaluation,  Time*Condition, Time*Overvaluation, **Condition*Overvaluation** | 9 | -11076 | -11008 | 5547 | -11094 | 0.23 | 0.634 |  | | 9 | 2839 | 2908 | -1411 | 2821 | 0.41 | 0.520 | |  |
| Time, Condition, Overvaluation,  Time*Condition, Time*Overvaluation, Condition*Overvaluation, **Time*Condition*Overvaluation** | 10 | -11095 | -11019 | 5558 | -11115 | 20.91 | 0.000 |  | | 10 | 2840 | 2916 | -1410 | 2820 | 1.58 | 0.209 | |  |

Note. Fixed effects in **bold** indicate the fixed effect added and tested for significance.

**Robustness Analyses**

**Children Robustness Analyses**

***Child Zygomaticus Activity***

Results are presented in Table S3. Robustness analyses replicated the findings of main analyses.

There were no main effects of condition or narcissism. There was a main effect of time, with zygomaticus activity decreasing over time. There was a two-way interaction, with zygomaticus activity decreasing over time for higher levels of narcissism in children. There was a three-way interaction between condition, narcissism, and time, with zygomaticus activity increasing over time for children higher in narcissism in the high-status condition.

We broke the three-way interaction down by condition. In the low-status condition, there was a two-way interaction between time and narcissism, with zygomaticus activity decreasing more steeply for children with higher narcissism levels, *β =* -.01, *SE* = .01, *t*(7149) = -2.53, *p* < .05. Zygomaticus activity decreased more steeply over time for children with high (*M* + 1*SD*) narcissism levels, *β =* -0.3, *SE* = .01, *p <* .001, than for children with low (*M* - 1*SD*) narcissism levels, *β = -*.01, *SE* = .01, *p =* 0.02. In the high-status condition, there was a two-way interaction between time and narcissism, with zygomaticus activity increasing more steeply for children with higher narcissism levels, *β =* .03, *SE* = .01, *t*(6970) = 4.66, *p* < .001. Zygomaticus activity remained stable for children with high (*M* + 1*SD*) narcissism levels, *β =* .00, *SE* = .01, *p =* .53, but decreased over time for children with low (*M* - 1*SD*) narcissism levels, *β = -*.02, *SE* = .01, *p < .*001. Together, these results show that children with higher levels of narcissism were inclined to smile decreasingly when losing status, but not when gaining status.

***Child Corrugator Activity***

Results are presented in Table S4. Robustness analyses replicated the findings of main analyses.

There were no main effects of condition or narcissism. There was a main effect of time, with corrugator activity increasing over time. There was a two-way interaction between time and narcissism, with corrugator activity increasing more steeply for children with higher narcissism levels. There was a three-way interaction between condition, narcissism, and time, with the increase of corrugator activity for children higher in narcissism being most pronounced in the high-status condition.

We broke the three-way interaction down by condition. In the low status condition, there was a two-way interaction between time and narcissism, with corrugator activity increasing more steeply for children with higher narcissism levels, *β =* .01, *SE* < .01, *t*(6797) = 2.91, *p* < .01. Corrugator activity increased more steeply for children with high (*M* + 1*SD*) narcissism levels, *β =* .04, *SE* < .01, *p <* .001, than for those with low (*M* – 1*SD*) narcissism levels, *β =* .03, *SE* < .01, *p <* .001. In the high-status condition, the effects of narcissism were similar but more pronounced. There was a two-way interaction between time and narcissism, with corrugator activity increasing more steeply for children with higher narcissism levels, *β =* .03, *SE* < .01, *t*(7024) = 9.32, *p* < .001. Corrugator activity increased more steeply for children with high (*M* + 1*SD*) narcissism levels, *β =* .07, *SE* < .01, *p <* .001, than for those with low (*M* – 1*SD*) narcissism levels, *β =* .02, *SE* < .01, *p <* .001. Together, these results show that children with higher levels of narcissism were most inclined to frown increasingly, especially so when gaining status.

**Parent Robustness Analyses**

***Parent Zygomaticus Activity, Children’s Narcissism***

Results are presented in Table S4. Robustness analyses replicated the findings of main analyses.

There were no main effects of children’s narcissism or condition. There was a main effect of time, with zygomaticus activity decreasing over time. There was a two-way interaction between condition and time, with zygomaticus activity increasing over time in the high-status condition. There was two-way interaction between time and narcissism, with zygomaticus activity decreasing over time for higher levels of narcissism in children. Finally, there was a three-way interaction between condition, children’s narcissism, and time, with zygomaticus activity increasing over time for parents of children with higher narcissism levels when children gained status.

We broke the 3-way interaction down by condition. In the low-status condition, there was a two-way interaction between time and children’s narcissism, with zygomaticus activity decreasing for higher levels of children’s narcissism, *β* > -.01, *SE* < .01, *t*(7307) = 2.87, p < .01. Zygomaticus activity decreased more for parents of children with high (M + 1SD) narcissism levels, *β* = -.01, *SE* < .01, p < .001, than for parents of children with low (M - 1SD) narcissism levels, *β* = -.02, *SE* < .01, p < .001. In the high-status condition, there was a two-way interaction between time and children’s narcissism, with zygomaticus activity increasing for higher levels of children’s narcissism, *β* = .01, *SE* < .01, *t*(7321) = 4.26, p < .001. Zygomaticus activity increased for parents of children with high (M + 1SD) narcissism, *β* = .01, *SE* < .01, p < .001, but it decreased for parents of children with low (M - 1SD) narcissism levels, *β* = -.01, *SE* < .01, p = .01. Together, these results show that parents of children with higher narcissism levels were most inclined to smile decreasingly when children lost status, but smiled increasingly when children gained status.

***Parent Corrugator Activity, Children’s Narcissism***

Results are presented in Table S3. While children’s narcissism did not moderate the effect of time on parents’ corrugator activity in main analyses, it did so in robustness analyses.

There were no main effects of condition or children’s narcissism. There was a main effect of time, with parents’ corrugator activity decreasing over time. There was a two-way interaction between time and condition, with corrugator activity increasing over time in the high-status condition. There was a two-way interaction between time and children’s narcissism, with corrugator activity increasing over time for parents of children with higher narcissism levels. Finally, there was a three-way interaction between children’s narcissism, condition, and time, with corrugator activity decreasing over time for parents of children with higher narcissism levels when children gained status. Thus, parents of children with higher narcissism levels were most inclined to frown decreasingly when children gained status.

We broke the three-way interaction down by condition. In the low-status condition, there was a two-way interaction between time and children’s narcissism, with parents’ corrugator activity increasing for higher narcissism levels in children, *β =* .01, *SE* < .01, *t*(7337) = -12.04, *p* < .001. Corrugator activity increased over time for parents of children with high (*M* + 1*SD*) narcissism levels, *β <* .01, *SE* < .01, *p <* .001, but decreased over time for parents of children with low (*M* - 1*SD*) narcissism levels, *β =* -.01, *SE* < .01, *p* < .001. In the high-status condition, there was no two-way interaction between time and children’s narcissism. Together, the results of robustness analyses show that parents of children with higher narcissism levels were most inclined to frown increasingly, whereas parents of children with lower narcissism levels were most inclined to frown decreasingly, when children lost status. Robustness analyses thus replicated and extended the results yielded by main analyses.

***Parent Zygomaticus Activity, Parental Overvaluation***

Results are presented in Table S5. Robustness analyses replicated the findings of main analyses.

There were no main effects of condition or parental overvaluation. There was a main effect of time, with zygomaticus activity decreasing over time. There was a two-way interaction between condition and time, with zygomaticus activity increasing over time in the high-status condition. Finally, there was a three-way interaction between condition, parental overvaluation and time, with zygomaticus activity increasing over time for more overvaluing parents when children gained status. Thus, more overvaluing parents were inclined to smile increasingly when children gained status.

We broke the 3-way interaction down by condition. In the low-status condition, there was no two-way interaction between time and parental overvaluation. In the high-status condition, there was a two-way interaction between time and parental overvaluation, with zygomaticus activity increasing for higher levels of parental overvaluation, *β* = .01, *SE* < .01, *t*(7321) = 9.29, p < .001. Zygomaticus activity increased for parents of children with high (M + 1SD) parental overvaluation levels, *β* = .01, *SE* < .01, p < .001, but it decreased for parents with low (M - 1SD) parental overvaluation levels, *β* = -.02, *SE* < .01, p < .001. Together, these results show that overvaluing parents were most inclined to smile increasingly when children gained status.

***Parent Corrugator Activity, Parental Overvaluation***

Results are presented in Table S5. While parental overvaluation did not moderate the effect of time on parents’ corrugator activity in main analyses, it did so in robustness analyses.

There were no main effects of condition or parental overvaluation. There was a main effect of time, with parents’ corrugator activity decreasing over time. There was a two-way interaction between time and condition, with corrugator activity increasing over time in the high-status condition. There was a two-way interaction between time and parental overvaluation, with corrugator activity increasing over time for more overvaluing parents. Finally, there was a three-way interaction between parental overvaluation, condition, and time, with corrugator activity decreasing over time for more overvaluing parents when children gained status. Thus, parents who overvalued their children more were most inclined to frown decreasingly when children gained status.

We broke the three-way interaction down by condition. In the low-status condition, there was a two-way interaction between time and parental overvaluation, with parents’ corrugator activity increasing for higher levels of parental overvaluation, *β <* .01, *SE* < .01, *t*(7337) = 5.77, *p* < .001. Corrugator activity remained stable for parents with high (*M* + 1*SD*) parental overvaluation levels, *β =* .00, *SE* = .00, *p* = .20, but decreased over time for parents with low (*M* - 1*SD*) parental overvaluation levels, *β =* -.01, *SE* < .01, *p* < .001. In the high-status condition, there was a two-way interaction between time and parental overvaluation, with parents’ corrugator activity decreasing for higher levels of parental overvaluation, *β >* -.01, *SE* < .01, *t*(7158) = 2.52, *p* < .05. Corrugator activity remained stable for parents with high (*M* + 1*SD*) parental overvaluation levels, *β =* .00, *SE* = .00, *p* = .13, but increased over time for parents with low (*M* - 1*SD*) parental overvaluation levels, *β <* .01, *SE* < .01, *p* < .001. Together, the results of robustness analyses show that less overvaluing parents were most inclined to frown decreasingly when children lost status and more inclined to frown increasingly when children gained status. By contrast, overvaluing parents did not exhibit changes in frowning over time in any of the conditions.

Table S3

*Robustness Analyses of Children’s Winsorized Muscle Activity Predicted by Condition, Time, and Children’s Narcissism Levels*

|  | Zygomaticus Activity | | |  | Corrugator Activity | | |
| --- | --- | --- | --- | --- | --- | --- | --- |
| Fixed Effects | *β* | *SE (β)* | *t* |  | *β* | *SE (β)* | *t* |
| Intercept | -.05 | .10 | -0.48 |  | -.08 | .08 | -0.96 |
| Condition | -.11 | .14 | -0.82 |  | -.19 | .12 | -1.61 |
| Time | -.02 | .00 | -5.39^***^ |  | .04 | .00 | 14.94^***^ |
| Narcissism | .07 | .09 | 0.72 |  | .05 | .08 | 0.66 |
| Condition*Time | .00 | .00 | 0.92 |  | .01 | .00 | 1.63 |
| Narcissism*Condition | .00 | .15 | 0.02 |  | -.03 | .12 | -0.28 |
| Time*Narcissism | -.01 | .00 | -2.51^*^ |  | .01 | .00 | 2.83^**^ |
| Condition*Time*Narcissism | .02 | .00 | 3.83^***^ |  | .02 | .00 | 5.40^***^ |

*Note*. Condition was dummy coded with “0” for Low Status and “1” for High Status.
* p < .05 ** p < .01 *** p < .001, *p* values calculated using Satterthwaite degrees of freedom.

Table S4

*Robustness Analyses of Parents’ Winsorized Muscle Activity Predicted by Condition, Time, and Children’s Narcissism Levels*

|  | Zygomaticus Activity | | |  | Corrugator Activity | | |
| --- | --- | --- | --- | --- | --- | --- | --- |
| Fixed Effects | *β* | *SE (β)* | *t* |  | *β* | *SE (β)* | *t* |
| Intercept | -.11 | .06 | -1.88 |  | -.14 | .03 | -4.77^***^ |
| Condition | .03 | .09 | 0.39 |  | -.02 | .04 | -0.46 |
| Time | -.01 | .00 | -8.19^***^ |  | -.03 | .00 | -4.57^***^ |
| Narcissism | .04 | .06 | 0.66 |  | -.01 | .03 | -0.27 |
| Condition*Time | .01 | .00 | 6.16^***^ |  | .01 | .00 | 6.01^***^ |
| Narcissism*Condition | -.12 | .09 | -1.40 |  | -.02 | .04 | -0.45 |
| Time*Narcissism | -.00 | .00 | -3.01^**^ |  | .01 | .00 | 12.19^***^ |
| Condition*Time*Narcissism | .01 | .00 | 5.02^***^ |  | -.01 | .00 | -8.46^***^ |

*Note*. Condition was dummy coded with “0” for Low Status and “1” for High Status.
* p < .05 ** p < .01 *** p < .001, *p* values calculated using Satterthwaite degrees of freedom.

Table S5

*Robustness Analyses of Parents’ Winsorized Muscle Activity Predicted by Condition, Time, and Parental Overvaluation Levels*

|  | Zygomaticus Activity | | |  | Corrugator Activity | | |
| --- | --- | --- | --- | --- | --- | --- | --- |
| Fixed Effects | *β* | *SE (β)* | *t* |  | *β* | *SE (β)* | *t* |
| Intercept | -.12 | 0.06 | -2.00^*^ |  | -.13 | .03 | -4.57^***^ |
| Condition | .06 | .09 | 0.67 |  | -.02 | .04 | -0.64 |
| Time | -.01 | .00 | -8.06^***^ |  | -.00 | .00 | -4.06^***^ |
| Overvaluation | -.03 | .06 | -0.50 |  | .01 | .03 | 0.43 |
| Condition*Time | .01 | .00 | 5.04^***^ |  | .01 | .00 | 5.91^***^ |
| Overvaluation*Condition | -.08 | .09 | -0.88 |  | .00 | .04 | 0.05 |
| Time*Overvaluation | -.01 | .00 | -0.77 |  | .00 | .00 | 5.86^***^ |
| Condition*Time*Overvaluation | .02 | .00 | 6.59^***^ |  | -.01 | .00 | -5.96^***^ |

*Note*. Condition was dummy coded with “0” for Low Status and “1” for High Status.
* p < .05 ** p < .01 *** p < .001, *p* values calculated using Satterthwaite degrees of freedom.

**Children Narcissistic Admiration and Rivalry Analyses**

Children also completed our adaptation for children of the six-item Narcissistic Admiration and Rivalry Questionnaire - Short form (NARQ-S; Back et al., 2013). The questionnaire measures, with three items each, an agentic narcissistic dimension termed *narcissistic admiration*, and an antagonistic narcissistic dimension termed *narcissistic rivalry*. The full scale can be found in the Supplementary Appendix A, below. Sample items include: “I enjoy thinking about how special I am” (admiration), and “If other children try to be better than me, I want them to fail” (rivalry). Items were rated on 4-point scales (0 = not agree at all, to 3 = agree completely). Responses were separately averaged across items for admiration (M = 0.78, SD = 0.47, α = .60) and rivalry (M = 0.67, SD= 0.57, α = .64), and jointly for the total scale (M = 0.89, SD= 0.63, α = .62). Admiration and rivalry were weakly, positively associated (*r*= .22, *p* = .05). As the scale has not been validated before in children, we refrained from including it in main analyses. We nevertheless repeated analyses with this instrument, to provide some first insights on how narcissistic admiration and rivalry moderate children’s affective responses to status gain and loss.

**Preliminary Analyses**

***Correlations with main variables***

Childhood narcissism measured by the CNS was highly associated with admiration (*r*= .62, *p* < .001) and the total NARQ-S score (*r*= .60, *p* = .001), and moderately associated with rivalry (*r*= .31, *p* < .05). Parental overvaluation was not significantly associated with any of the NARQ-S narcissism indicators.

***Random Assignment***

There were no condition differences in children’s admiration, *t*(81)= 0.10, *p* = .90, rivalry, *t*(81)= 0.90, *p* = .40, or total admiration and rivalry score, *t*(81)= 0.61, *p* = .50. Children’s corrugator and zygomaticus activity during the baseline did not differ by children’s admiration levels. However, children’s corrugator activity during the baseline differed by narcissistic rivalry, with higher overall corrugator activity for children higher in rivalry, *β* = .71, *SE* = .18, *t*(83)= 3.93, *p* < .001, and lower overall corrugator activity for children higher in rivalry when children would gain status, *β* = -.63, *SE* = .21, *t*(83)= -2.93, *p* < .01. Breakdown by condition revealed a positive effect of rivalry in low status, *β* = .71, *SE* = .22, *t*(42)= 3.25, *p* < .01, but no effect of rivalry in high status. Likewise, corrugator activity differed by total narcissistic admiration and rivalry scores, with higher overall corrugator activity for children higher in rivalry, *β* = .46, *SE* = .16, *t*(83)= 2.80, *p* < .01, and lower overall corrugator activity for children higher in rivalry when children would gain status, *β* = -.49, *SE* = .21, *t*(83)= -2.32, *p* < .01. Breakdown by condition revealed a positive effect of rivalry in low status, *β* = .46, *SE* = .20, *t*(42)= 2.29, *p* = .03, but no effect of rivalry in high status. When analyzing the effect of narcissistic rivalry and the effect of total admiration and rivalry on children’s corrugator activity during the popularity game, we controlled for baseline corrugator activity to take into account these preexisting baseline differences.

**Children Narcissistic Admiration Analyses**

***Child Zygomaticus Activity***

Results are presented in Table S6. There were no main effects of condition or narcissistic admiration. There was a main effect of time, with zygomaticus activity decreasing over time. There were no two-way interactions. There was a three-way interaction between condition, time, and narcissistic admiration, with zygomaticus activity increasing over time for children higher in narcissistic admiration when children gained status.

We broke the three-way interaction down by condition. In the low-status condition, there was no two-way interaction between time and narcissistic admiration. In the high-status condition, there was an interaction between narcissistic admiration and time, with zygomaticus activity increasing over time for children higher in narcissistic admiration, *β* = .01, *SE* = .01, *t*(7337) = 2.37, *p* < .05. Zygomaticus activity remained stable for children with high (M + 1SD) narcissistic admiration levels, *β* = .00, *SE* = .01, *p* = .98, but decreased over time for children with low (M - 1SD) narcissistic admiration levels, *β* = -.02, *SE* = .01, *p* < .001. Together, these results show that only children low in narcissistic admiration were inclined to smile decreasingly when gaining status.

***Child Corrugator Activity***

Results are presented in Table S6. There were no effects of condition or narcissistic admiration. There was a main effect of time, with corrugator activity increasing over time. There was a two-way interaction between time and condition, witch corrugator activity increasing more steeply in the high-status condition. There was a two-way interaction between time and narcissism, with corrugator activity increasing more steeply for children with higher narcissistic admiration levels. Thus, children with higher levels of narcissistic admiration were inclined to frown increasingly, both when losing, and when gaining status.

**Children Narcissistic Rivalry Analyses**

***Child Zygomaticus Activity***

Results are presented in Table S7. There was no main effect of condition or narcissistic rivalry. There was a main effect of time, with zygomaticus activity decreasing over time. There was a two-way interaction between time and narcissistic rivalry, with zygomaticus activity decreasing more steeply for children higher in narcissistic rivalry. There was a three-way interaction between condition, time, and narcissistic rivalry, with zygomaticus activity increasing over time for children higher in narcissistic rivalry when children gained status.

We broke the 3-way interaction down by condition. In the low-status condition, there was a two-way interaction between time and narcissistic rivalry, with zygomaticus activity decreasing more steeply for children with higher narcissistic rivalry levels, *β* = -.03, *SE* = .01, *t*(7516) =-5.46, *p* < .001. Zygomaticus activity decreased over time for children with high (*M* + 1*SD*) narcissistic rivalry levels, *β* = -.06, *SE* = .01, *p* < .001, but it remained stable for children with low (*M* - 1*SD*) narcissistic rivalry levels, *β* = .01, *SE* < .01, *p* = 0.25. In the high-status condition, there was a two-way interaction between time and narcissistic rivalry, with zygomaticus activity increasing for children with higher narcissistic rivalry levels, *β* =.01, *SE* < .01, *t*(7337) = 2.02, *p* = .04. Zygomaticus activity remained stable over time for children with high (*M* + 1*SD*) narcissistic rivalry levels, *β* = .00, *SE* = .01, *p* < .54, but decreased over time for children with low (*M* - 1*SD*) narcissistic rivalry levels, *β* = -.02, *SE* = .01, *p* < 0.001. Together, these results show that children with high levels of rivalry were inclined to smile decreasingly when losing status, whereas children with low levels of rivalry were inclined to smile decreasingly when gaining status.

***Child Corrugator Activity***

Results are presented in Table S7. There was a main effect of condition, with corrugator activity being lower in the high-status condition. There was a main effect of time, with corrugator activity increasing over time. There was a two-way interaction between condition and time, with corrugator activity increasing more steeply in the high-status condition. There was a two-way interaction between time and narcissistic rivalry, with corrugator activity decreasing over time for children higher in narcissistic rivalry. There was a three-way interaction between condition, time, and narcissistic rivalry, with corrugator activity increasing over time for children higher in narcissistic rivalry when children gained status.

We broke the 3-way interaction down by condition. In the low-status condition, there was a two-way interaction between time and narcissistic rivalry, with corrugator activity decreasing over time for children with higher narcissistic rivalry levels, *β* = -.02, *SE* < .01, *t*(7516) =-5.85, *p* < .001. Corrugator activity remained stable for children with high (*M* + 1*SD*) narcissistic rivalry levels, *β* = .00, *SE* < .01, *p* = .91, but it increased over time for children with low (*M* - 1*SD*) narcissistic rivalry levels, *β* = .04, *SE* < .01, *p* < .001. In the high-status condition, there was no two-way interaction between time and narcissistic rivalry. Together, these results show that only the children with low rivalry were most inclined to frown increasingly when losing status.

**Children Narcissistic Admiration and Rivalry Total Score Analyses**

***Child Zygomaticus Activity***

Results are presented in Table S8. There were no main effects of condition or narcissism. There was a main effect of time, with zygomaticus activity decreasing over time. There was a two-way interaction between time and narcissism, with zygomaticus activity decreasing over time for children higher in narcissism. There was a three-way interaction between condition, narcissism, and time, with zygomaticus activity increasing over time for children higher in narcissism in the high-status condition.

We broke the three-way interaction down by condition. In the low-status condition, there was a two-way interaction between time and narcissism, with zygomaticus activity decreasing over time for children higher in narcissism, *β =* -.02, *SE* = .01, *t*(7516) = -3.86, *p* < .001. Zygomaticus activity decreased over time for children with high (*M* + 1*SD*) narcissism levels, *β =* -.04, *SE* = .01, *p <* .001, but remained stable over time for children with low (*M* - 1*SD*) narcissism levels, *β =* -*.*00, *SE* = .01, *p = .*75. In the high-status condition, there was a two-way interaction between time and narcissism, with zygomaticus activity increasing for children with higher narcissism levels, *β =* .01, *SE* < .01, *t*(7337) = 2.77, *p* = .01. Zygomaticus activity remained stable over time for children with high (*M* + 1*SD*) narcissism levels, *β =* .00, *SE* = .01, *p =* .96, but decreased over time for children with low (*M* - 1*SD*) narcissism levels, *β = -*.03, *SE* = .01, *p < .*001. Together, these results show that children with higher narcissism levels were most inclined to smile decreasingly when losing status, but not when gaining status.

***Child Corrugator Activity***

Results are presented in Table S8. There was a main effect of condition, with corrugator activity being lower in the high-status condition. There was a main effect of time with corrugator activity increasing over time. There was a two-way interaction between time and condition, with corrugator activity increasing over time in the high-status condition. There was a three-way interaction between condition, time, and narcissism, with corrugator activity increasing over time for children with higher narcissism levels in the high-status condition.

We broke the three-way interaction down by condition. In the low-status condition, the interaction between narcissism and time was insignificant. In the high-status condition, there was a two-way interaction between time and narcissism, with corrugator activity increasing over time for children with higher narcissism levels, *β* = .01, *SE* < .01, *t*(7337) =3.79, *p* < .001. Corrugator activity increased more steeply for children with high (*M* + 1*SD*) narcissism levels, *β =* .06, *SE* < .01, *p <* .001, than for those with low (*M* – 1*SD*) narcissism levels, *β =* .04, *SE* < .01, *p <* .001. Together, these results show that children with higher narcissism levels were most inclined to frown increasingly when gaining status.

**Children Narcissistic Admiration Robustness Analyses**

***Child Zygomaticus Activity***

Results are presented in Table S9. Findings from robustness analyses differed from findings of main analyses.

There were no main effects of condition or narcissistic admiration. There was a main effect of time, with zygomaticus activity decreasing over time. There were no two-way or three-way interactions. Thus, robustness analyses did not replicate findings from main analyses, showing no effect of narcissistic admiration on children’s zygomaticus activity.

***Child Corrugator Activity***

Results are presented in Table S9. Findings from robustness analyses replicated findings from main analyses.

There were no main effects of condition or narcissistic admiration. There was a main effect of time, with corrugator activity increasing over time. There was a two-way interaction between time and condition, with corrugator activity increasing over time in the high-status condition. There was a two-way interaction between time and narcissistic admiration, with corrugator activity increasing over time for children with higher levels of narcissistic admiration. Thus, children with higher levels of narcissistic admiration were inclined to frown increasingly, both when losing, and when gaining status.

**Children Narcissistic Rivalry Robustness Analyses**

***Child Zygomaticus Activity***

Results are presented in Table S10. Findings from robustness analyses replicated findings of main analyses.

There was no main effect of condition or narcissistic rivalry. There was a main effect of time, with zygomaticus activity decreasing over time. There was a two-way interaction between time and narcissistic rivalry, with zygomaticus activity decreasing more steeply for children higher in narcissistic rivalry. There was a three-way interaction between condition, time, and narcissistic rivalry, with zygomaticus activity increasing over time for children higher in narcissistic rivalry when children gained status.

We broke the three-way interaction down by condition. In the low-status condition, there was a two-way interaction between time and narcissistic rivalry, with zygomaticus activity decreasing more steeply for children with higher narcissistic rivalry levels, *β* = -.03, *SE* < .01, *t*(7149) =-7.29, *p* < .001. Zygomaticus activity decreased over time for children with high (*M* + 1*SD*) narcissistic rivalry levels, *β* = -.05, *SE* = .01, *p* < .001, but increased over time for children with low (*M* - 1*SD*) narcissistic rivalry levels, *β* = .01, *SE* < .01, *p* = 0.04. In the high-status condition, there was a two-way interaction between time and narcissistic rivalry, with zygomaticus activity increasing for children with higher narcissistic rivalry levels, *β* =.01, *SE* < .01, *t*(7192) = 3.82, *p* < .001. Zygomaticus activity remained stable over time for children with high (*M* + 1*SD*) narcissistic rivalry levels, *β* = .00, *SE* < .01, *p* = .44, but decreased over time for children with low (*M* - 1*SD*) narcissistic rivalry levels, *β* = -.03, *SE* < .01, *p* < .001. Together, these results show that children with high levels of rivalry were inclined to smile decreasingly when losing status, whereas children with low levels of rivalry were inclined to smile decreasingly when gaining status.

***Child Corrugator Activity***

Results are presented in Table S10. Findings from robustness analyses differed from findings of main analyses.

There was a main effect of condition, with corrugator activity being lower in the high-status condition. There was a main effect of time, with corrugator activity increasing over time. There were no two-way or three-way interactions. Thus, robustness analyses did not replicate findings from main analyses, showing no effect of narcissistic rivalry on children’s zygomaticus activity.

**Children Narcissistic Admiration and Rivalry Total Score Robustness Analyses**

***Child Zygomaticus Activity***

Results are presented in Table S11. Findings from robustness analyses replicated findings from main analyses.

There were no main effects of condition or narcissism. There was a main effect of time, with zygomaticus activity decreasing over time. There was a two-way interaction between time and narcissism, with zygomaticus activity decreasing over time for children higher in narcissism. There was a three-way interaction between condition, narcissism, and time, with zygomaticus activity increasing over time for children higher in narcissism in the high-status condition.

We broke the three-way interaction down by condition. In the low-status condition, there was a two-way interaction between time and narcissism, with zygomaticus activity decreasing over time for children higher in narcissism, *β =* -.01, *SE* < .01, *t*(7149) = -3.59, *p* < .001. Zygomaticus activity decreased over time for children with high (*M* + 1*SD*) narcissism levels, *β =* -.03, *SE* = .01, *p <* .001, but remained stable over time for children with low (*M* - 1*SD*) narcissism levels, *β =* -*.*01, *SE* < .01, *p = .*31. In the high-status condition, there was a two-way interaction between time and narcissism, with zygomaticus activity increasing for children with higher narcissism levels, *β =* .01, *SE* = .01, *t*(6970) = 2.95, *p* < .01. Zygomaticus activity remained stable over time for children with high (*M* + 1*SD*) narcissism levels, *β = -*.01, *SE* < .01, *p =* .15, but decreased over time for children with low (*M* - 1*SD*) narcissism levels, *β = -*.02, *SE* < .01, *p < .*001. Together, these results show that children with higher narcissism levels were most inclined to smile decreasingly when losing status, but not when gaining status.

***Child Corrugator Activity***

Results are presented in Table S11. Findings from robustness analyses on the conditional effect of narcissism partly differed from findings of main analyses, showing higher increase in corrugator activity over time in the low status condition for children with higher narcissism levels.

There was a main effect of condition, with corrugator activity being lower in the high-status condition. There was a main effect of time, with corrugator activity increasing over time. There was a two-way interaction between time and condition, with corrugator activity increasing over time in the high-status condition. There was a three-way interaction between condition, time, and narcissism, with corrugator activity decreasing over time for children with higher narcissism levels in the high-status condition.

We broke the three-way interaction down by condition. In the low-status condition, there was a two-way interaction between time and narcissism, with corrugator activity increasing over time for children with higher narcissism levels, *β* = .02, *SE* < .01, *t*(6799) =5.93, *p* < .001. Corrugator activity increased more steeply for children with high (*M* + 1*SD*) narcissism levels, *β =* .06, *SE* < .01, *p <* .001, than for those with low (*M* – 1*SD*) narcissism levels, *β =* .02, *SE* < .01, *p <* .001. In the high-status condition, there was a two-way interaction between time and narcissism, with corrugator activity increasing over time for children with higher narcissism levels, *β* = .01, *SE* < .01, *t*(7025) =3.34, *p* < .001. Corrugator activity increased more steeply for children with high (*M* + 1*SD*) narcissism levels, *β =* .05, *SE* < .01, *p <* .001, than for those with low (*M* – 1*SD*) narcissism levels, *β =* .04, *SE* < .01, *p <* .001. Together, these results show that children with higher narcissism levels were most inclined to frown increasingly, and especially so when losing status.

**Integrative Discussion of Findings From CNS and NARQ**

Our supplementary analyses parse narcissism into narcissistic admiration and rivalry. Children with higher levels of narcissistic admiration most strongly mirrored children with higher levels of CNS narcissism on corrugator activity: they frowned increasingly when both gaining and losing status. Thus, these children seemed to experience stress during status pursuit, regardless of whether this pursuit was successful or unsuccessful. By contrast, children with higher levels of narcissistic rivalry most strongly mirrored children with higher levels of CNS narcissism on zygomaticus activity: they increasingly smiled when gaining status. Given that narcissistic rivalry represents the antagonistic dimension of narcissism (Back et al., 2013; Grapsas, Brummelman, Back, & Denissen, 2020). This finding suggests that children with higher levels of narcissistic rivalry possibly enjoyed their status supremacy over their competitors.

Table S6

*Analyses of Children’s Winsorized Muscle Activity Predicted by Condition, Time, and Children’s Narcissistic Admiration Levels*

|  | Zygomaticus Activity | | |  | Corrugator Activity | | |
| --- | --- | --- | --- | --- | --- | --- | --- |
| Fixed Effects | *β* | *SE (β)* | *t* |  | *β* | *SE (β)* | *t* |
| Intercept | .11 | .14 | 0.79 |  | .19 | .16 | 1.28 |
| Condition | -.22 | .20 | -1.13 |  | -.38 | .21 | -1.81 |
| Time | -.02 | .01 | -4.38^***^ |  | .02 | .00 | 8.34^***^ |
| Admiration | -.05 | .14 | -0.36 |  | .17 | .15 | 1.14 |
| Condition*Time | .01 | .01 | -1.36 |  | .03 | .00 | 8.22^***^ |
| Admiration *Condition | .04 | .20 | 0.20 |  | -.27 | .21 | -1.31 |
| Time*Admiration | -.01 | .01 | -1.22 |  | .01 | .00 | 3.49^***^ |
| Condition*Time*Admiration | .02 | .01 | 2.62^**^ |  | .00 | .00 | 0.54 |

*Note*. Condition was dummy coded with “0” for Low Status and “1” for High Status.
* p < .05 ** p < .01 *** p < .001, *p* values calculated using Satterthwaite degrees of freedom.

Table S7

*Analyses of Children’s Winsorized Muscle Activity Predicted by Condition, Time, and Children’s Narcissistic Rivalry Levels*

|  | Zygomaticus Activity | | |  | Corrugator Activity | | |
| --- | --- | --- | --- | --- | --- | --- | --- |
| Fixed Effects | *β* | *SE (β)* | *t* |  | *β* | *SE (β)* | *t* |
| Intercept | .12 | .14 | 0.86 |  | .07 | .04 | 1.48 |
| Condition | -.24 | .20 | -1.22 |  | -.13 | .07 | -2.04^*^ |
| Time | -.03 | .01 | -5.00^***^ |  | .02 | .00 | 7.47^***^ |
| Rivalry | .10 | .18 | 0.53 |  | .06 | .06 | 0.96 |
| Condition*Time | .01 | .01 | 1.71 |  | .03 | .00 | 8.66^***^ |
| Rivalry*Condition | -.01 | .22 | -0.06 |  | -.04 | .07 | -0.53 |
| Time*Rivalry | -.03 | .01 | -5.16^***^ |  | -.02 | .00 | -5.86^***^ |
| Condition*Time*Rivalry | .04 | .01 | 5.49^***^ |  | .02 | .00 | 5.85^***^ |

*Note*. Condition was dummy coded with “0” for Low Status and “1” for High Status. Corrugator analyses control for baseline corrugator activity.

* p < .05 ** p < .01 *** p < .001, *p* values calculated using Satterthwaite degrees of freedom.

Table S8

*Analyses of Children’s Winsorized Muscle Activity Predicted by Condition, Time, and Children’s Narcissistic Admiration and Rivalry (NARQ) Levels*

|  | Zygomaticus Activity | | |  | Corrugator Activity | | |
| --- | --- | --- | --- | --- | --- | --- | --- |
| Fixed Effects | *β* | *SE (β)* | *t* |  | *β* | *SE (β)* | *t* |
| Intercept | .11 | .14 | 0.79 |  | .06 | .05 | 1.41 |
| Condition | -.23 | .20 | -1.15 |  | -.13 | .07 | -1.99^*^ |
| Time | -.02 | .01 | -4.63^***^ |  | .02 | .00 | 8.23^***^ |
| NARQ | .00 | .16 | 0.00 |  | .05 | .05 | 0.87 |
| Condition*Time | .01 | .01 | 1.44 |  | .03 | .00 | 8.11^***^ |
| NARQ*Condition | .05 | .20 | 9.25 |  | -.01 | .07 | -0.17 |
| Time*NARQ | -.02 | .01 | -3.64^***^ |  | -.00 | .00 | -0.46 |
| Condition*Time*NARQ | .03 | .01 | 4.67^***^ |  | .01 | .00 | 2.71^**^ |

*Note*. Condition was dummy coded with “0” for Low Status and “1” for High Status. Corrugator analyses control for baseline corrugator activity.

* p < .05 ** p < .01 *** p < .001, *p* values calculated using Satterthwaite degrees of freedom.

Table S9

*Robustness Analyses of Children’s Winsorized Muscle Activity Predicted by Condition, Time, and Children’s Narcissistic Admiration Levels*

|  | Zygomaticus Activity | | |  | Corrugator Activity | | |
| --- | --- | --- | --- | --- | --- | --- | --- |
| Fixed Effects | *β* | *SE (β)* | *t* |  | *β* | *SE (β)* | *t* |
| Intercept | -.05 | .10 | -0.53 |  | -.09 | .08 | -1.03 |
| Condition | -.11 | .14 | -0.75 |  | -.18 | .12 | -1.54 |
| Time | -.02 | .00 | -5.27^***^ |  | .04 | .00 | 15.08^***^ |
| Admiration | -.10 | .10 | -0.97 |  | -.03 | .09 | -0.36 |
| Condition*Time | .00 | .00 | 0.98 |  | .01 | .00 | 2.00^*^ |
| Admiration *Condition | .08 | .14 | 0.56 |  | .02 | .12 | 0.17 |
| Time*Admiration | .00 | .00 | 0.33 |  | .02 | .00 | 6.09^***^ |
| Condition*Time*Admiration | -.00 | .00 | -0.33 |  | -.00 | .00 | -0.73 |

*Note*. Condition was dummy coded with “0” for Low Status and “1” for High Status.
* p < .05 ** p < .01 *** p < .001, *p* values calculated using Satterthwaite degrees of freedom.

Table S10

*Robustness Analyses of Children’s Winsorized Muscle Activity Predicted by Condition, Time, and Children’s Narcissistic Rivalry Levels*

|  | Zygomaticus Activity | | |  | Corrugator Activity | | |
| --- | --- | --- | --- | --- | --- | --- | --- |
| Fixed Effects | *β* | *SE (β)* | *t* |  | *β* | *SE (β)* | *t* |
| Intercept | -.05 | .10 | -0.47 |  | -.96 | .08 | -12.01^***^ |
| Condition | -.12 | .14 | -0.84 |  | -.14 | .06 | -2.32^*^ |
| Time | -.02 | .00 | -6.29^***^ |  | .04 | .00 | 14.56^***^ |
| Rivalry | .03 | .13 | 0.26 |  | .04 | .06 | 0.62 |
| Condition*Time | .01 | .00 | 1.53 |  | .01 | .00 | 1.95 |
| Rivalry*Condition | .04 | .16 | 0.25 |  | -.02 | .07 | -0.22 |
| Time*Rivalry | -.03 | .00 | -7.22^***^ |  | .01 | .00 | 1.86 |
| Condition*Time*Rivalry | .04 | .01 | 8.16^***^ |  | -.01 | .00 | -1.42 |

*Note*. Condition was dummy coded with “0” for Low Status and “1” for High Status.

Corrugator analyses control for baseline corrugator activity.

* p < .05 ** p < .01 *** p < .001, *p* values calculated using Satterthwaite degrees of freedom.

Table S11

*Robustness Analyses of Children’s Winsorized Muscle Activity Predicted by Condition, Time, and Children’s Narcissistic Admiration and Rivalry (NARQ) Levels*

|  | Zygomaticus Activity | | |  | Corrugator Activity | | |
| --- | --- | --- | --- | --- | --- | --- | --- |
| Fixed Effects | *β* | *SE (β)* | *t* |  | *β* | *SE (β)* | *t* |
| Intercept | -.06 | .10 | -0.56 |  | .04 | .04 | 0.89 |
| Condition | -.11 | .14 | -0.75 |  | -.14 | .06 | -2.31^*^ |
| Time | -.02 | .00 | -5.55^***^ |  | .04 | .00 | 15.57^***^ |
| NARQ | -.07 | .12 | -0.59 |  | .03 | .05 | .054 |
| Condition*Time | .01 | .00 | 1.10 |  | .01 | .00 | 1.42 |
| NARQ*Condition | .11 | .15 | 0.74 |  | .01 | .06 | .015 |
| Time*NARQ | -.01 | .00 | -3.56^***^ |  | .02 | .00 | 5.77^***^ |
| Condition*Time*NARQ | .02 | .00 | 4.28^***^ |  | -.01 | .00 | -2.60^**^ |

*Note*. Condition was dummy coded with “0” for Low Status and “1” for High Status.

Corrugator analyses control for baseline corrugator activity.

* p < .05 ** p < .01 *** p < .001, *p* values calculated using Satterthwaite degrees of freedom.

**Children’s Self-Esteem Analyses**

To examine the specificity of our findings, we included self-esteem in our study. Developmental research shows that narcissism and self-esteem are modestly associated because they both involve favorable self-views, but they are also distinct because the quality of the favorable self-views they involve differs (Brummelman & Sedikides, 2020; Brummelman, Thomaes, & Sedikides, 2016). Narcissism reflects a sense of superiority and entitlement, whereas self-esteem reflects a sense of worth as a person. Whereas narcissism increases risk for psychopathology (e.g., conduct problems), self-esteem protects against it (e.g., lower anxiety and depression; Harris, Donnellan, & Trzesniewski, 2018). To test the specificity of findings in children, we repeated narcissism analyses controlling for their self-esteem levels, and then we conducted with self-esteem the same analyses we conducted with narcissism.

**Measure of Self-Esteem**

We measured self-esteem with the four-item Lifespan Self-Esteem Scale (LSES; Harris et al., 2018). Sample items include: “How do you feel about yourself?”, and “How do you feel about the kind of person you are?”. Items were rated on 5-point scales (1 = Really sad, to 5 = Really Happy), with each response option accompanied by an illustration of the corresponding feeling (e.g., Really Sad is illustrated with a crying face, Really Happy is illustrated with an open-mouthed smile). Responses were averaged across items (*M* = 4.06, *SD* = 0.58, Cronbach’s *a* = .80). Like with narcissism, for multilevel analyses, we z-standardized self-esteem scores.

**Correlation With Children’s Narcissism**

Supporting their conceptual distinction, narcissism and self-esteem were only modestly positively correlated in our final sample, *r*(81) = .24, p = .029.

**Narcissism Analyses Controlling for Self-Esteem**

We repeated analyses on the effect of children’s narcissism on their muscle activity while controlling for children’s self-esteem levels. Results remained virtually unchanged, and this was also confirmed by the lack of significant differences between the models with and without self-esteem as a covariate (zygomaticus *χ*^2^ (1) = 0.28, *p* = .600; corrugator *χ*^2^ (1) = 0.04, *p* = .833). For parsimony, we omit the full presentation of these results.

**Effects of Children’s Self-Esteem on Muscle Activity**

We conducted in children the same analyses for self-esteem that we conducted for narcissism. Results are presented in Table S12.

***Child Zygomaticus Activity***

Zygomaticus activity analyses for self-esteem mirrored those of narcissism. There was a main effect of time, with zygomaticus activity decreasing over the course of the experiment. There were no two-way interactions, but there was a three-way interaction between condition, time, and self-esteem.

We broke the three-way interaction down by condition. In the low-status condition, there was no interaction between time and self-esteem. In the high-status condition, however, there was a significant two-way interaction between time and self-esteem, *β* = .03, *SE* = .01, *t*(7339) =5.60, *p* < .001. Zygomaticus activity increased over time for children with high (*M* + 1*SD*) self-esteem, *β* = .02, *SE* = .01, *p* = .02, but decreased over time for children with low *M* - 1*SD*) self-esteem, *β* = -.05, *SE* = .01, *p* < .001. Thus, like children with high narcissism, children with high self-esteem increasingly smiled when gaining status. Like children with low narcissism, children with low self-esteem decreasingly smiled when gaining status.

***Child Corrugator Activity***

Corrugator activity analyses for self-esteem mirrored those of narcissism when children gained status but were opposite when children lost status. There was a main effect of time, with corrugator activity increasing over time. There were two-way interactions between time and condition, as well as between time and self-esteem. These interactions, however, were qualified by a three-way interaction between time, condition, and self-esteem.

We broke down the three-way interaction by condition. In the low status condition, there was a significant two-way interaction between time and self-esteem, *β* = -.01, *SE* < .01, *t*(7518) = -2.77, *p* = .006. Corrugator activity increased less steeply for children with high (*M* + 1*SD*) self-esteem, *β* = .01, *SE* < .01, *p* < .001, than for children with low (*M* - 1*SD*) self-esteem *β* = .03, *SE* < .01, *p* < .001. In the high-status condition, there was also a significant two-way interaction between time and self-esteem, *β* =.01, *SE* < .01, *t*(7339) = 4.40, *p* < .001. Corrugator activity increased more steeply for children with high (*M* + 1*SD*) self-esteem, *β* = .06, *SE* < .01, *p* < .001, than for children with low (*M* - 1*SD*) self-esteem *β* = .04, *SE* < .01, *p* < .001. Thus, like children with high narcissism, children with high self-esteem increasingly smiled when gaining status. However, unlike children with high narcissism, children with high self-esteem smiled less when gaining status.

**Discussion of Self-Esteem Analyses**

The results from self-esteem analyses showed that effects of narcissism on muscle activity were not owed to children’s self-esteem levels. Furthermore, they showed that narcissism and self-esteem have partly similar and partly different effects on children’s muscle activity. When gaining status, children with higher self-esteem seemed to experience the more intense mixture of positive and negative affect experienced by children with high narcissism. By contrast, when losing status, children with lower self-esteem seemed to experience the more intense negative affect experienced by children with higher narcissism levels. These results show that, unlike narcissism, children’s self-esteem protects them from the negative impact of status loss, but is, like narcissism, responsible, for children’s heightened affective ambivalence during status gain. The results also suggest that the overall pattern of findings attributed to narcissism seems distinct, and thus not owed to an underlying transdiagnostic, pathological trait.

Table S12

*Analyses of Children’s Winsorized Muscle Activity Predicted by Condition, Time, and Children’s Self-Esteem Levels*

|  | Zygomaticus Activity | | |  | Corrugator Activity | | |
| --- | --- | --- | --- | --- | --- | --- | --- |
| Fixed Effects | *β* | *SE (β)* | *t* |  | *β* | *SE (β)* | *t* |
| Intercept | 0.10 | 0.14 | 0.73 |  | 0.18 | 0.15 | 1.21 |
| Condition | -0.21 | 0.20 | -1.03 |  | -0.39 | 0.21 | -1.86 |
| Time | -0.02 | 0.01 | -4.18^***^ |  | 0.02 | 0.00 | 7.77^***^ |
| Self-Esteem | -0.05 | 0.13 | -0.36 |  | -0.04 | 0.14 | -0.29 |
| Condition*Time | 0.00 | 0.01 | 0.48 |  | 0.03 | 0.00 | 7.88^***^ |
| Condition*Self-Esteem | 0.00 | 0.20 | -0.02 |  | 0.19 | 0.21 | 0.87 |
| Time* Self-Esteem | 0.00 | 0.00 | 0.87 |  | -0.01 | 0.00 | -2.78^***^ |
| Condition*Time* Self-Esteem | 0.03 | 0.01 | 3.96^***^ |  | 0.02 | 0.00 | 5.15^***^ |

*Note*. Condition was dummy coded with “0” for Low Status and “1” for High Status.

* p < .05 ** p < .01 *** p < .001, *p* values calculated using Satterthwaite degrees of freedom.

**Children’s Affective Synchrony Analyses**

When gaining status, narcissism in children was related to increases in both frowning and smiling. To investigate whether these increases were synchronous (i.e., if children with higher narcissism simultaneously experienced increases in positive and negative affect), we regressed children’s zygomaticus activity on corrugator activity, condition, narcissism, and all their possible interactions (controlling for baseline zygomaticus activity).

Results are presented in Table S13. There was a positive effect of baseline, but no effects of condition, corrugator activity, or narcissism. There was a two-way interaction between corrugator activity and condition. However, this was qualified by a three-way interaction between corrugator activity, condition, and narcissism.

We broke down the three-way interaction by condition. In the low status condition, there was no two-way interaction between narcissism and corrugator activity. In the high-status condition, however, there was a significant two-way interaction between narcissism and corrugator activity. Corrugator activity was unrelated to zygomaticus activity for children with high (*M +* 1*SD*) narcissism, *β* = 0.01, *SE* = 0.03, *p* = .641. However, corrugator activity was positively related to zygomaticus activity for children with low (*M -* 1*SD*) narcissism, *β* = 0.88, *SE* = 0.03, *p* < .001. Thus, when gaining status, for children with high narcissism, smiling and frowning did not co-occur. In conjunction with findings from main analyses, this suggests that children with high narcissism did not experience increases in positive and negative affect at the same time. Instead, they might have experienced fluctuations from heightened positive to heightened negative affect, though future studies should test this assumption.

Table S13

*Analyses of Children’s Winsorized Zygomaticus Activity Predicted by Baseline, Condition, Corrugator Activity, and Children’s Narcissism Levels*

|  | Zygomaticus Activity | | |
| --- | --- | --- | --- |
| Fixed Effects | *β* | *SE (β)* | *t* |
| Intercept | -0.90 | 0.09 | -10.01^***^ |
| Baseline Zygomaticus | 109.93 | 6.54 | 16.80^***^ |
| Corrugator Activity | 0.02 | 0.02 | 0.98 |
| Condition | -0.04 | 0.10 | -0.39 |
| Narcissism | 0.07 | 0.06 | 1.08 |
| Corrugator Activity*Condition | 0.43 | 0.03 | 14.68^***^ |
| Corrugator Activity*Narcissism | -0.02 | 0.02 | -0.93 |
| Condition*Narcissism | -0.14 | 0.10 | -1.40 |
| Condition*Corrugator Activity*Narcissism | -0.41 | 0.03 | -14.18^***^ |

*Note*. Condition was dummy coded with “0” for Low Status and “1” for High Status.

* p < .05 ** p < .01 *** p < .001, *p* values calculated using Satterthwaite degrees of freedom.

**Cue Reactivity Analyses**

To delve deeper into our findings, we examined whether the reactivity patterns we found in children and parents were owed to differences in their reactivity toward cues of status gain (i.e., likes children received) and loss (i.e., likes fictitious competitors received).

**Reactivity to Likes Toward Self (Status Gain Cues)**

First, we analyzed participants’ responses to the likes children received. We first created a dummy variable in the data indicating the first three seconds following each like (coded as 1), because muscle activity in response to circumscribed cues usually peaks within 3 seconds (Dufner et al., 2015).

***Children***

We regressed, in separate models, zygomaticus and corrugator activity on the likes dummy, on experimental condition, on narcissism, and on all their possible interactions, while controlling for baseline muscle activity (Table S14). All focal effects were non-significant in both models.

***Parents***

We regressed, in separate models, zygomaticus and corrugator activity on the likes dummy, on experimental condition, on children’s narcissism or overvaluation, and on all their possible interactions, while controlling for baseline muscle activity (Tables S15 & S16). In both zygomaticus activity models, all focal effects were non-significant. In both corrugator activity models, there was a significant two-way interaction between likes and condition, whereas all other effects were non-significant. Probing these two-way interactions in both models showed that, in the low status condition, parents frowned marginally more when their children received a like (narcissism model *β* = .03, *SE* = 0.02, t = 1.89, *p =* 0.06; overvaluation model *β* = .03, *SE* = 0.02, t = 1.89, *p =* 0.05). However, in both models, this difference in parents’ corrugator reactivity was absent in the high status condition (narcissism model *β* = -.01, *SE* = 0.01, t = -0.88, *p =* 0.38; overvaluation model *β* = -.01, *SE* = 0.01, t = -0.89, *p =* 0.36.

***Summary***

The results suggest that children’s reactions to the task were not owed to how much they smiled or frowned in response to the individual likes they received. Like their children, parents did not smile more in response to the individual likes their children received. However, parents frowned marginally more when children received likes in the low status condition (perhaps a sign of displeasure because these likes did not change their child’s status), but this was not related to children’s narcissism, or to parental overvaluation levels. Therefore, the main results regarding narcissism and parental overvaluation do not appear driven by individual differences in status gain cue reactivity.

**Reactivity to Likes Toward Competitors (Status Loss Cues)**

We tried to analyze participants’ responses to likes others received. Because such likes were heavily dispersed in both experimental conditions and often quickly followed each other (e.g., often occurring one or two seconds after another) over the course of the task, isolating children’s responses to such likes was impossible.

Table S14

*Analyses of Children’s Winsorized Muscle Activity Predicted by Condition, Likes, and Children’s Narcissism Levels*

|  | Zygomaticus Activity | | |  | Corrugator Activity | | | |
| --- | --- | --- | --- | --- | --- | --- | --- | --- |
| Fixed Effects | *β* | *SE (β)* | *t* |  | *β* | | *SE (β)* | *t* |
| Intercept | -0.92 | 0.07 | -13.32^***^ | |  | -0.16 | 0.19 | -0.85 |
| Baseline | 112.81 | 5.02 | 22.47^***^ |  | 38.93 | | 13.93 | 2.79^**^ |
| Likes | -0.01 | 0.03 | -0.39 |  | 0.01 | | 0.01 | 0.83 |
| Condition | -0.11 | 0.07 | -1.44 |  | -0.34 | | 0.21 | -1.64 |
| Narcissism | 0.07 | 0.05 | 1.50 |  | 0.16 | | 0.13 | 1.17 |
| Likes*Condition | 0.01 | 0.03 | 0.37 |  | -0.03 | | 0.02 | -1.76 |
| Likes*Narcissism | 0.03 | 0.03 | 1.20 |  | 0.02 | | 0.01 | 1.60 |
| Condition*Narcissism | -0.06 | 0.08 | -0.75 |  | -0.21 | | 0.21 | -1.01 |
| Likes*Condition*Narcissism | -0.02 | 0.03 | -0.51 |  | -0.01 | | 0.02 | -0.68 |

*Note*. Condition was dummy coded with “0” for Low Status and “1” for High Status. Like was dummy coded with “1” for muscle activity corresponding to the first 3 seconds following each like children received, and with “0” for all other timepoints.

* p < .05 ** p < .01 *** p < .001, *p* values calculated using Satterthwaite degrees of freedom.

Table S15

*Analyses of Parents’ Winsorized Muscle Activity Predicted by Condition, Likes, and Children’s Narcissism Levels*

|  | Zygomaticus Activity | | |  | Corrugator Activity | | |
| --- | --- | --- | --- | --- | --- | --- | --- |
| Fixed Effects | *β* | *SE (β)* | *t* |  | *β* | *SE (β)* | *t* |
| Intercept | -0.70 | 0.03 | -22.10^***^ |  | -0.42 | 0.05 | -7.64^***^ |
| Baseline | 80.56 | 1.62 | 49.68^***^ |  | 32.52 | 1.32 | 24.58^***^ |
| Likes | -0.02 | 0.01 | -1.77 |  | 0.03 | 0.02 | 1.89 |
| Condition | -0.05 | 0.04 | -1.35 |  | 0.11 | 0.07 | 1.45 |
| Narcissism | 0.00 | 0.03 | -0.05 |  | -0.02 | 0.05 | -0.39 |
| Likes*Condition | 0.02 | 0.01 | 1.49 |  | -0.04 | 0.02 | -2.09^*^ |
| Likes*Narcissism | 0.01 | 0.01 | 0.87 |  | 0.00 | 0.02 | 0.01 |
| Condition*Narcissism | -0.01 | 0.04 | -0.23 |  | 0.02 | 0.08 | 0.25 |
| Likes*Condition*Narcissism | -0.01 | 0.01 | -0.75 |  | 0.00 | 0.02 | 0.16 |

*Note*. Condition was dummy coded with “0” for Low Status and “1” for High Status. Like was dummy coded with “1” for muscle activity corresponding to the first 3 seconds following each like children received, and with “0” for all other timepoints.

* p < .05 ** p < .01 *** p < .001, *p* values calculated using Satterthwaite degrees of freedom.

Table S16

*Analyses of Parents’ Winsorized Muscle Activity Predicted by Condition, Likes, and Parental Overvaluation Levels*

|  | Zygomaticus Activity | | |  | Corrugator Activity | | |
| --- | --- | --- | --- | --- | --- | --- | --- |
| Fixed Effects | *β* | *SE (β)* | *t* |  | *β* | *SE (β)* | *t* |
| Intercept | -0.70 | 0.03 | -21.78^***^ |  | -0.42 | 0.06 | -7.49^***^ |
| Baseline | 80.66 | 1.62 | 49.70^***^ |  | 32.50 | 1.32 | 24.53^***^ |
| Likes | -0.02 | 0.01 | -1.45 |  | 0.03 | 0.02 | 1.92 |
| Condition | -0.06 | 0.04 | -1.45 |  | 0.11 | 0.08 | 1.46 |
| Overvaluation | 0.01 | 0.03 | 0.28 |  | -0.01 | 0.05 | -0.15 |
| Likes*Condition | 0.01 | 0.01 | 1.18 |  | -0.04 | 0.02 | -2.13^*^ |
| Likes* Overvaluation | 0.02 | 0.01 | 1.66 |  | 0.01 | 0.02 | 0.31 |
| Condition*Overvaluation | 0.01 | 0.04 | 0.13 |  | 0.00 | 0.08 | -0.02 |
| Likes*Condition* Overvaluation | -0.02 | 0.01 | -1.37 |  | 0.00 | 0.02 | -0.11 |

*Note*. Condition was dummy coded with “0” for Low Status and “1” for High Status. Like was dummy coded with “1” for muscle activity corresponding to the first 3 seconds following each like children received, and with “0” for all other timepoints.

* p < .05 ** p < .01 *** p < .001, *p* values calculated using Satterthwaite degrees of freedom.

**Analyses of Demographic Effects on Main Findings in Children**

**Children’s Gender Effects**

Given that there are documented gender differences in narcissism and children’s social goals, we examined whether children’s gender moderated our findings. We first repeated analyses controlling for children’s gender. Then, we repeated analyses including a four-way interaction term in each of the children analyses. When this interaction was significant, we performed main analyses separately per gender.

***Controlling for Gender***

We repeated analyses on the effect of children’s narcissism on their muscle activity while controlling for children’s gender. Results remained virtually unchanged, and this was also confirmed by the lack of significant differences between the models with and without gender as a covariate (zygomaticus *χ*^2^ (1) = 1.06, *p* = .303; corrugator *χ*^2^ (1) = 0.89, *p* = .346). For parsimony, we omit the full presentation of these results.

***Gender as Moderator, Zygomaticus Activity.***

We repeated analyses on the effect of children’s narcissism on their zygomaticus activity by including gender and all its possible interactions with other variables. This resulted in a model with a four-way interaction including time, condition, narcissism, and gender, which was significant, *β =* 0.15, *SE* = 0.02, *t*(14849) = 8.98, *p <* .001. We therefore repeated main analyses separately for girls and boys.

**Girls Model*.*** Results are presented in Table S17. There were no main effects or two-way interactions. However, there was a significant three-way interaction between condition, time, and narcissism.

We broke down the three-way interaction by condition. In the low-status condition, there was no two-way interaction between time and narcissism. In the high-status condition, however, there was a two-way interaction between time and narcissism, *β* = .08, *SE* = .01, *t*(3399) = 7.98, *p* <.001. Zygomaticus activity increased for girls with high (*M +* 1*SD*) narcissism, *β =* .06, *SE = .*01, *p* < .001, but decreased for girls with low (*M* - 1*SD*) narcissism, *β = -*.11, *SE = .*01, *p* < .001. Therefore, girls with high narcissism increasingly smiled when gaining status.

**Boys Model*.*** Results are presented in Table S18. There was an effect of time, with zygomaticus activity decreasing over time. There was a two-way interaction between condition and time, and between time and narcissism, yet these were qualified by a three-way interaction between condition, time, and narcissism.

We broke the three-way interaction down by condition. In the low-status condition, there was a two-way interaction between time and narcissism, *β* = .05, *SE* = .01, *t*(3578) = 7.08, *p* <.001. Zygomaticus activity remained stable for boys with high (*M +* 1*SD*) narcissism, *β =* .01, *SE = .*01, *p* = .471, but decreased for boys with low (*M* - 1*SD*) narcissism, *β = -*.07, *SE = .*01, *p* < .001. In the high-status condition, the interaction between time and narcissism was non-significant. Therefore, boys with low narcissism decreasingly smiled when losing status.

***Gender as Moderator, Corrugator Activity***

We repeated analyses on the effect of children’s narcissism on their corrugator activity by including gender and all its possible interactions with other variables. This resulted in a model with a four-way interaction including time, condition, narcissism, and gender, which was significant, *β =* 0.02, *SE* = 0.01, *t*(14849) = 2.02, *p =* .043. We therefore repeated main analyses separately for girls and boys.

**Girls Model.** Results are presented in Table S17. There were no condition, time, or narcissism main effects. There were two-way interactions between condition and time, as well as between time and narcissism. However, these were qualified by a three-way interaction between condition, narcissism, and time.

We broke down the three-way interaction by condition. In the low-status condition, there was a two-way interaction between time and narcissism, *β =* .01, *SE < .*01, t(3936) = 3.55, *p <.*001. Corrugator activity increased for girls with high (*M +* 1*SD*) narcissism, *β =* .01, *SE = .*01, *p* = .005, but decreased for girls with low (*M* - 1*SD*) narcissism, β = -.01, SE = 0.01, p = .047. In the high-status condition, there was also a two-way interaction between time and narcissism, *β* = .05, *SE* = .01, *t*(3399) = 98, *p* <.001. Corrugator activity increased for girls with high (*M* + 1*SD*) narcissism, β = .04, SE < .01, p < .001, but remained stable for girls with low (*M* - 1 *SD*) narcissism, β = -.01, SE = 0.01, p = .159. Therefore, girls with higher narcissism increasingly frowned, and especially when gaining status.

**Boys Model.** Results are presented in Table S18. There was a main effect of time, with corrugator activity increasing over time. There was a two-way interaction between condition and time, as well as a two-way interaction between time and narcissism. These were qualified, however, by a three-way interaction between condition, time, and narcissism.

We broke down the three-way interaction by condition. In the low-status condition, there was a two-way interaction between time and narcissism, *β =* -.02, *SE < .*01, t(3578) = -3.82, *p <.*001. Corrugator activity increased less steeply for boys with high (*M +* 1*SD*) narcissism, *β =* .03, *SE = .*01, *p <* .001, but more steeply for boys with low (*M* - 1 *SD*) narcissism, *β =* .06, *SE = .*01, *p <* .001. In the high-status condition, however, the interaction between time and narcissism was not significant. Therefore, boys with high narcissism

***Summary of Gender Effects***

Main results were virtually unchanged when controlling for gender. Including gender as a moderator revealed that the effects of narcissism were more pronounced in girls than in boys. Given the limited sample size and the complexity of such interactions, these findings should be replicated in future research.

**Children’s Age Effects**

Given that there are documented age differences in narcissism and children’s social goals, we examined whether children’s age moderated our findings. We first repeated analyses controlling for children’s age. Then, we repeated analyses including a four-way interaction term with age in each of the children analyses. When this interaction was significant, we performed main analyses separately per age group (*M* - 1*SD* of age /*M* + 1*SD* of age).

***Controlling for Age***

We repeated analyses on the effect of children’s narcissism on their muscle activity while controlling for children’s age. Results remained virtually unchanged, and this was also confirmed by the lack of significant differences between the models with and without age as a covariate (zygomaticus *χ*^2^ (1) = 0.18, *p* = .673; corrugator *χ*^2^ (1) = 0.43, *p* = .511). For parsimony, we omit the full presentation of these results.

***Age as Moderator, Zygomaticus Activity***

We repeated analyses on the effect of children’s narcissism on their zygomaticus activity by including age and all its possible interactions with other variables. This resulted in a model with a four-way interaction including time, condition, narcissism, and age, which was significant, *β =* 0.02, *SE* = 0.01, *t*(14849) = 2.52, *p =* .012. We therefore repeated main analyses separately for younger (age ≥ *M +* 1*SD*) and older (age ≤ *M -* 1*SD*) children.

**Younger Children Model.** Results are presented in Table S19. There were no main effects or two-way interactions, but there was a three-way interaction between time, condition, and narcissism.

We broke the three-way interaction down by condition. In the low-status condition, there was no two-way interaction between time and narcissism. In the high-status condition, however, there was a two-way interaction between time and narcissism, *β* = .06, *SE* = .01, *t*(3399) = 6.50, *p* <.001. Zygomaticus activity increased for younger children with high (*M +* 1*SD*) narcissism, *β =* .05, *SE = .*01, *p* < .001, but decreased for younger children with low (*M* - 1*SD*) narcissism, *β = -*.07, *SE = .*01, *p* < .001. Therefore, younger children with high narcissism increasingly smiled when gaining status.

**Older Children Model.** Results are presented in Table S19. There was a main effect of time, with zygomaticus activity decreasing over time. There was a two-way interaction between condition and time, as well as a two-way interaction between time and narcissism. There was no three-way interaction between condition, time, and narcissism.

We broke down the two-way interaction between condition and time by condition. In the low-status condition, there was an increase in zygomaticus activity over time, *β* = .02, *SE* = .01, *p* = .020. In the high-status condition, there was a decrease in zygomaticus activity over time, *β* = -.06, *SE* = .01, *p* < .001. Thus, older children in the low-status condition increasingly smiled when losing status, but decreasingly smiled when gaining status.

We also broke down the two-way interaction between time and narcissism by narcissism levels. Zygomaticus activity decreased more steeply over time for older children with high (*M +* 1*SD*) narcissism, *β* = -.04, *SE* = .01, *p* < .001, than for older children with low (*M -* 1*SD*) narcissism, *β* = -.02, *SE* = .01, *p* = .025. Thus, older children with high narcissism decreasingly smiled in both conditions.

***Age as Moderator, Corrugator Activity***

We repeated analyses on the effect of children’s narcissism on their corrugator activity by including age and all its possible interactions with other variables. This resulted in a model with a four-way interaction including time, condition, narcissism, and age, which was not statistically significant, *β = -*0.01, *SE* < .01, *t*(14849) = -1.94, *p =* .052. We therefore did not repeat main analyses separately for younger (age ≥ *M +* 1*SD*) and older (age ≤ *M -* 1*SD*) children.

***Summary of Age Effects***

Main results were virtually unchanged when controlling for age. Including age as a moderator revealed that the effects of narcissism on zygomaticus activity were more pronounced in younger children, whereas there were no differences in the effects of narcissism between younger and older children. Given the limited sample size and the complexity of such interactions, these findings should be replicated in future research.

**Parental Education Effects**

Given that there are documented class differences in narcissism and children’s social goals, we examined whether parents’ education level, an important indicator of social class, moderated children’s affective reactivity. Parents reported their highest level of education, with 11 categories (1= lower, basic education to 11 = scientific education (bachelor, masters and above). Assuming a linear relationship between parent education level and children’s affective reactivity to social status, we included parents’ education level as a continuous (*M =* 8.66, *SD* = 2.61), z-standardized variable in the analyses. We regressed, in separate models, children’s zygomaticus and corrugator activity on experimental condition, on time, on parental education, and on all their possible interactions. Results are presented in Table S20.

***Zygomaticus Activity***

There was a main effect of time, with zygomaticus decreasing over time, and a main effect of education level, with zygomaticus associated with lower education level. There was also a two-way interaction between time and education, yet it was qualified by a three-way interaction between condition, time, and education level.

We broke down the three-way interaction by condition. In the low-status condition, the interaction between time and education level was significant, *β* = 0.04, *SE* < .01, *t*(7516) – 9.24, *p* < .001. Zygomaticus activity increased for children of higher (*Max* value) educated parents, *β =* 0.02, *SE* = .01, *p =* .009, but decreased for children of lower (*M –* 1*SD*) educated parents, *β =* -0.63, *SE* = .01, *p <* .001. In the high-status condition, there was no two-way interaction between time and education level. Therefore, when children lost status, children of higher educated parents increasingly smiled, whereas children of lower educated parents decreasingly smiled.

***Corrugator Activity***

There was a main effect of time, with corrugator increasing over time. There was a two-way interaction between condition and time, and a two-way interaction between time and education, yet these were qualified by a three-way interaction between condition, time, and education level.

We broke down the three-way interaction by condition. In the low-status condition, the interaction between time and education level was significant, *β* = 0.01, *SE* < .01, *t*(7516) = 5.49, *p* < .001. Corrugator activity increased more steeply for children of higher (*Max* value) educated parents, *β =* 0.03, *SE* < .01, *p <* .001 than for children of lower (*M –* 1*SD*) educated parents, *β =* 0.01, *SE* < .01, *p =* .022. In the high-status condition, there was no two-way interaction between time and education level. Therefore, when children lost status, children of higher educated parents frowned more strongly over time than children of less educated parents.

***Summary***

These analyses suggest that children from higher-educated parents frowned and smiled more in response to status loss. There were no effects involving parental education level when children gained status.

Table S17

*Analyses of Girls’ Winsorized Muscle Activity Predicted by Condition, Time, and Narcissism*

|  | Zygomaticus Activity | | |  | Corrugator Activity | | |
| --- | --- | --- | --- | --- | --- | --- | --- |
| Fixed Effects | *β* | *SE (β)* | *t* |  | *β* | *SE (β)* | *t* |
| Intercept | 0.36 | 0.24 | 1.49 |  | 0.29 | 0.26 | 1.11 |
| Condition | -0.51 | 0.36 | -1.41 |  | -0.38 | 0.39 | -0.96 |
| Time | -0.01 | 0.01 | -1.62 |  | 0.01 | 0.00 | 1.49 |
| Narcissism | 0.12 | 0.19 | 0.65 |  | 0.10 | 0.21 | 0.50 |
| Condition*Time | 0.01 | 0.01 | 0.89 |  | 0.05 | 0.01 | 8.57^***^ |
| Condition* Narcissism | -0.08 | 0.37 | -0.21 |  | -0.07 | 0.41 | -0.16 |
| Time* Narcissism | -0.01 | 0.01 | -1.43 |  | 0.01 | 0.00 | 3.71^***^ |
| Condition*Time*Narcissism | 0.09 | 0.01 | 7.46^***^ |  | 0.04 | 0.01 | 6.07^***^ |

*Note*. Condition was dummy coded with “0” for Low Status and “1” for High Status.

* p < .05 ** p < .01 *** p < .001, *p* values calculated using Satterthwaite degrees of freedom.

Table S18

*Analyses of Boys’ Winsorized Muscle Activity Predicted by Condition, Time, and Narcissism*

|  | Zygomaticus Activity | | |  | Corrugator Activity | | |
| --- | --- | --- | --- | --- | --- | --- | --- |
| Fixed Effects | *β* | *SE (β)* | *t* |  | *β* | *SE (β)* | *t* |
| Intercept | -0.08 | 0.17 | -0.46 |  | 0.03 | 0.18 | 0.20 |
| Condition | -0.01 | 0.23 | -0.04 |  | -0.29 | 0.25 | -1.20 |
| Time | -0.05 | 0.01 | -7.08^***^ |  | 0.05 | 0.00 | 12.92^***^ |
| Narcissism | -0.18 | 0.20 | -0.87 |  | 0.30 | 0.21 | 1.41 |
| Condition*Time | 0.05 | 0.01 | 5.42^***^ |  | 0.01 | 0.01 | 2.32^*^ |
| Condition* Narcissism | 0.22 | 0.26 | 0.83 |  | -0.34 | 0.28 | -1.21 |
| Time* Narcissism | 0.05 | 0.01 | 5.70^***^ |  | -0.02 | 0.00 | -3.54^***^ |
| Condition*Time*Narcissism | -0.05 | 0.01 | -5.18^***^ |  | 0.02 | 0.01 | 3.51^***^ |

*Note*. Condition was dummy coded with “0” for Low Status and “1” for High Status.

* p < .05 ** p < .01 *** p < .001, *p* values calculated using Satterthwaite degrees of freedom.

Table S19

*Analyses of Younger* (*M – 1SD) Children’s Winsorized Muscle Activity Predicted by Condition, Time, and Narcissism*

|  | Younger Children | | |  | Older Children | | |
| --- | --- | --- | --- | --- | --- | --- | --- |
| Fixed Effects | *β* | *SE (β)* | *t* |  | *β* | *SE (β)* | *t* |
| Intercept | 0.02 | 0.24 | 0.10 |  | 0.20 | 0.26 | 0.80 |
| Condition | -0.01 | 0.01 | -1.60 |  | -0.45 | 0.40 | -1.12 |
| Time | 0.04 | 0.33 | 0.11 |  | -0.06 | 0.01 | -9.45^***^ |
| Narcissism | -0.17 | 0.20 | -0.84 |  | -0.14 | 0.28 | -0.51 |
| Condition*Time | 0.01 | 0.01 | 0.65 |  | 0.08 | 0.01 | 7.87^***^ |
| Condition* Narcissism | 0.00 | 0.01 | -0.62 |  | -0.19 | 0.75 | -0.25 |
| Time* Narcissism | 0.29 | 0.31 | 0.95 |  | -0.02 | 0.01 | -2.78^**^ |
| Condition*Time*Narcissism | 0.06 | 0.01 | 5.80^***^ |  | 0.02 | 0.02 | 1.14 |

*Note*. Condition was dummy coded with “0” for Low Status and “1” for High Status.

* p < .05 ** p < .01 *** p < .001, *p* values calculated using Satterthwaite degrees of freedom.

Table S20

*Analyses of Children’s Winsorized Muscle Activity Predicted by Condition, Time, and Parental Education Level*

|  | Zygomaticus Activity | | |  | Corrugator Activity | | |
| --- | --- | --- | --- | --- | --- | --- | --- |
| Fixed Effects | *β* | *SE (β)* | *t* |  | *β* | *SE (β)* | *t* |
| Intercept | 0.10 | 0.14 | 0.71 |  | 0.19 | 0.15 | 1.26 |
| Condition | -0.21 | 0.19 | -1.07 |  | -0.38 | 0.21 | -1.77 |
| Time | -0.02 | 0.01 | -4.03^***^ |  | 0.02 | 0.00 | 8.50^***^ |
| Education | -0.35 | 0.13 | -2.65^**^ |  | 0.10 | 0.15 | 0.65 |
| Condition*Time | 0.01 | 0.01 | 1.17 |  | 0.03 | 0.00 | 8.15^***^ |
| Condition* Education | 0.30 | 0.19 | 1.55 |  | -0.11 | 0.21 | -0.49 |
| Time* Education | 0.04 | 0.00 | 8.71^***^ |  | 0.01 | 0.00 | 5.50^***^ |
| Condition*Time* Education | -0.05 | 0.01 | -6.88^***^ |  | -0.02 | 0.00 | -4.59^***^ |

*Note*. Condition was dummy coded with “0” for Low Status and “1” for High Status.

* p < .05 ** p < .01 *** p < .001, *p* values calculated using Satterthwaite degrees of freedom.

**Supplementary Appendices**

**Supplementary Appendix A**

Table S21

*Complete List of Self-Report Questionnaires Included in the Study*

| Name | Measure Type | Rater | Part of Procedure |
| --- | --- | --- | --- |
| Demographics (constructed for this study) | - | Children, Parents | Pre-Task |
| Lifespan Self-esteem Scale (Harris et al., 2018) | Trait | Children, Parents | Pre-Task |
| Childhood Narcissism Scale (Thomaes, Stegge, Bushman, Olthof, & Denissen, 2008) | Trait | Children, Parents | Pre-Task |
| Narcissistic Admiration and Rivalry Questionnaire – Short Form (Back et al., 2013; adapted for children for this study) | Trait | Children, Parents | Pre-Task |
| Narcissistic Personality Inventory – 16 (Ames, Rose, & Anderson, 2006) | Trait | Parents | Pre-Task |
| Parental Overvaluation Scale (Brummelman, Thomaes, Nelemans, Orobio de Castro, & Bushman, 2015) | Trait | Parents | Pre-Task |
| Status Motive Scale (constructed for this study) | Trait | Children, Parents | Pre-Task |
| State Shame and Guilt Scale,  Pride Subscale ((Brummelman et al., 2013)Marschall, Sanftner, & Tangney, 1994) | State | Children, Parents | Post-Task |
| State Shame and Guilt Scale,  Shame Subscale (Marschall et al., 1994) | State | Children, Parents | Post-Task |
| State Anger Scale (Laurent et al., 1999) | State | Children, Parents | Post-Task |
| State Anxiety Scale (items from Positive and Negative Affect Schedule; Laurent et al., 1999) | State | Children, Parents | Post-Task |
| State Depression Scale (items from Positive and Negative Affect Schedule; Laurent et al., 1999) | State | Children, Parents | Post-Task |
| State Hostility Scale (constructed for this study) | State | Children | Post-Task |
| Status Motive Item (constructed for this study) | State | Children, Parents | Post-Task |
| Inclusion of Child in the Self Scale (Aron, Aron, & Smollan, 1992; Brummelman, Thomaes, Slagt, Overbeek, De Castro, & Bushman, 2013) | State | Parents | Post-Task |
| Message From Child to Peers (constructed for this study) | Open-ended question | Children | Post-Task |
| Message From Parent to Child (constructed for this study) | Open-ended question | Parents | Post-Task |
| Manipulation Check Scales (constructed for this study) | - | Children, Parents | Post-Task |
| Fidelity Check (constructed for this study) | Open-ended questions | Children, Parents | Post-Task |

**Supplementary Appendix B**

***Adaptation of the NARQ-S for Children***

The original NARQ-S (Back et al., 2013) is designed to measure narcissistic admiration and rivalry in adolescents and adults and has, to our knowledge, not been used in research with children. To make the content of the scale more accessible and relevant for children, three of the manuscript’s authors adapted the items of the English Scale, translated the items to Dutch, and back to English. Below, the reader can find both the Dutch adaptation, which was used in the study, and the back-translation to English, which can be used as a point of reference to the original NARQ-S.

**NARQ-S, Child Version in Dutch.**

Kies het antwoord dat het best bij je mening past.

Je hebt een antwoordformaat met 4 categorieën ter beschikking (van 0 = „Helemaal niet waar“ tot 3 = „Helemaal waar“).

|  | Helemaal niet waar | Niet echt waar | Best wel waar | Helemaal waar |
| --- | --- | --- | --- | --- |
| 1. Ik reageer bozig als andere kinderen meer aandacht krijgen dan ik. | 0 | 1 | 2 | 3 |
| 2. Ik verdien speciale aandacht | 0 | 1 | 2 | 3 |
| 3. Als andere kinderen proberen beter te zijn dan ik, dan hoop ik dat het ze mislukt. | 0 | 1 | 2 | 3 |
| 4. Ik vind het fijn om na te denken over hoe speciaal ik ben. | 0 | 1 | 2 | 3 |
| 5. Ik krijg veel aandacht omdat ik speciale dingen kan. | 0 | 1 | 2 | 3 |
| 6. De meeste andere kinderen zijn sukkels. | 0 | 1 | 2 | 3 |

**NARQ-S, English Translation of the Child Version in Dutch.**

Please circle the answer that fits your opinion best.

You have a response format with 4 options ranging from “0 = Not at all true” to “3 = Completely true”.

|  | Not at all true | Not really true | Quite true | Completely true |
| --- | --- | --- | --- | --- |
| 1. I react angrily if other children get more attention than I | 0 | 1 | 2 | 3 |
| 2. I deserve special attention | 0 | 1 | 2 | 3 |
| 3. If other children are trying to be better than me, I hope they fail at this | 0 | 1 | 2 | 3 |
| 4. I find it nice to think about how special I am | 0 | 1 | 2 | 3 |
| 5. I get a lot of attention because I can do special things | 0 | 1 | 2 | 3 |
| 6. Most other children are losers | 0 | 1 | 2 | 3 |

***NARQ-S, Child Version, Scoring Instructions***

The total NARQ-S score is calculated by averaging scores across all Items. The admiration subscale score is calculated by averaging the scores of Items 2, 4, and 5. The rivalry subscale score is calculated by averaging the scores of Items 1, 3, and 6.

Supplementary References

Ames, D. R., Rose, P., & Anderson, C. P. (2006). The NPI-16 as a short measure of narcissism. *Journal of Research in Personality*, *40*(4), 440–450.

Aron, A., Aron, E. N., & Smollan, D. (1992). Inclusion of other in the self scale and the structure of interpersonal closeness. *Journal of Personality and Social Psychology*, *63*(4), 596–612.

Back, M. D., Küfner, A. C. P., Dufner, M., Gerlach, T. M., Rauthmann, J. F., & Denissen, J. J. A. (2013). Narcissistic admiration and rivalry: Disentangling the bright and dark sides of narcissism. *Journal of Personality and Social Psychology*, *105*(6), 1013–1037. https://doi.org/10.1037/a0034431

Brummelman, E., & Sedikides, C. (2020). Raising children with high self-esteem (but not narcissism). *Child Development Perspectives*, *14*(2), 83–89. https://doi.org/10.1111/cdep.12362

Brummelman, E., Thomaes, S., & Sedikides, C. (2016). Separating narcissism from self-esteem. *Current Directions in Psychological Science*, *25*(1), 8–13. https://doi.org/10.1177/0963721415619737

Brummelman, E., Thomaes, S., Nelemans, S. A., Orobio de Castro, B., & Bushman, B. J. (2015). My child is God’s gift to humanity: Development and validation of the Parental Overvaluation Scale (POS). *Journal of Personality and Social Psychology*, *108*, 665–679. doi:10.1037/pspp0000012

Brummelman, E., Thomaes, S., Slagt, M., Overbeek, G., de Castro, B. O., & Bushman, B. J. (2013). My Child Redeems My Broken Dreams: On Parents Transferring Their Unfulfilled Ambitions onto Their Child. *PLoS ONE*, *8*(6), e65360. https://doi.org/10.1371/journal.pone.0065360

Brummelman, E., Thomaes, S., Slagt, M., Overbeek, G., De Castro, B. O., & Bushman, B. J. (2013). My child redeems my broken dreams: On parents transferring their unfulfilled ambitions onto their child. *PloS one, 8*(6), e65360.

Dufner, M., Arslan, R. C., Hagemeyer, B., Schönbrodt, F. D., & Denissen, J. J. A. (2015). Affective contingencies in the affiliative domain: Physiological assessment, associations with the affiliation motive, and prediction of behavior. *Journal of Personality and Social Psychology*, *109*(4), 662–676. https://doi.org/10.1037/pspp0000025

Fridlund, A. J., & Cacioppo, J. T. (1986). Guidelines for human electromyographic research. *Psychophysiology*, *23*(5), 567-589.

Grapsas, S., Brummelman, E., Back, M. D., & Denissen, J. J. A. (2020). The “why” and “how” of narcissism: A process model of narcissistic status pursuit. *Perspectives on Psychological Science: A Journal of the Association for Psychological Science*, *15*(1), 150–172. https://doi.org/10.1177/1745691619873350

Harris, M. A., Donnellan, M. B., & Trzesniewski, K. H. (2018). The Lifespan Self-Esteem Scale: Initial validation of a new measure of global self-esteem. *Journal of personality assessment*, *100*(1), 84-95.

Laurent, J., Catanzaro, S. J., Joiner, T. E., Jr., Rudolph, K. D., Potter, K. I., Lambert, S., . . . Gathright, T. (1999). A measure of positive and negative affect for children: Scale development and preliminary validation. *Psychological Assessment*, *11*, 326–338. doi:10.1037/1040-3590.11.3.326

Marschall, D., Sanftner, J., & Tangney, J. P. (1994). The State Shame and Guilt Scale. Fairfax, VA: George Mason University.

Spielberger, C. D. (1988). *Manual for the State-Trait Anger Expression Inventory (STAXI)*. Odessa, FL: Psychological Assessment Resources.

Thomaes, S., Stegge, H., Bushman, B. J., Olthof, T., & Denissen, J. (2008). Development and validation of the Childhood Narcissism Scale. *Journal of Personality Assessment*, *90*(4), 382–391. https://doi.org/10.1080/00223890802108162

Thomaes, S., Stegge, H., Bushman, B. J., Olthof, T., & Denissen, J. J. A. (2008). Development and validation of the Childhood Narcissism Scale. *Journal of Personality Assessment*, *90*(4), 382–391.

Van Boxtel, A. (2010). Facial EMG as a tool for inferring affective states. In A. J. Spink, F. Grieco, O. Krips, L. Loijens, L. Noldus, & P. Zimmerman (Eds.), *Proceedings of Measuring Behavior 2010* (pp. 104-108). Wageningen: Noldus Information technology.
